# Chronic SIV-Induced neuroinflammation disrupts CCR7^+^ CD4^+^ T cell immunosurveillance in the rhesus macaque brain

**DOI:** 10.1172/JCI175332

**Published:** 2024-03-12

**Authors:** Sonny R. Elizaldi, Chase E. Hawes, Anil Verma, Yashavanth Shaan Lakshmanappa, Ashok R. Dinasarapu, Brent T. Schlegel, Dhivyaa Rajasundaram, Jie Li, Blythe P. Durbin-Johnson, Zhong-Min Ma, Pabitra B. Pal, Danielle Beckman, Sean Ott, Reben Raeman, Jeffrey Lifson, John H. Morrison, Smita S. Iyer

**Affiliations:** 1 Graduate Group in Immunology, UCD, Davis, California, USA.; 2 Department of Pathology, School of Medicine, University of Pittsburgh, Pennsylvania, USA.; 3 California National Primate Research Center, UCD, Davis, California, USA.; 4 Department of Neurology, School of Medicine, Emory University, Atlanta, Georgia, USA.; 5 Department of Pediatrics, School of Medicine, University of Pittsburgh, Pittsburgh, Pennsylvania, USA.; 6 Bioinformatics Core, UCD, Davis, California, USA.; 7 AIDS and Cancer Virus Program, Frederick National Laboratory, Frederick, Maryland, USA.; 8 Department of Neurology, School of Medicine, and; 9 Department of Pathology, Microbiology, and Immunology, School of Veterinary Medicine, UCD, Davis, California, USA.

**Keywords:** AIDS/HIV, Inflammation, Adaptive immunity, Neurological disorders, T cells

## Abstract

CD4^+^ T cells survey and maintain immune homeostasis in the brain, yet their differentiation states and functional capabilities remain unclear. Our approach, combining single-cell transcriptomic analysis, ATAC-Seq, spatial transcriptomics, and flow cytometry, revealed a distinct subset of CCR7^+^ CD4^+^ T cells resembling lymph node central memory (T_CM_) cells. We observed chromatin accessibility at the *CCR7*, *CD28*, and *BCL-6* loci, defining molecular features of T_CM_. Brain CCR7^+^ CD4^+^ T cells exhibited recall proliferation and interleukin-2 production ex vivo, showcasing their functional competence. We identified the skull bone marrow as a local niche for these cells alongside CNS border tissues. Sequestering T_CM_ cells in lymph nodes using FTY720 led to reduced CCR7^+^ CD4^+^ T cell frequencies in the cerebrospinal fluid, accompanied by increased monocyte levels and soluble markers indicating immune activation. In macaques chronically infected with SIVCL757 and experiencing viral rebound due to cessation of antiretroviral therapy, a decrease in brain CCR7^+^ CD4^+^ T cells was observed, along with increased microglial activation and initiation of neurodegenerative pathways. Our findings highlight a role for CCR7^+^ CD4^+^ T cells in CNS immune surveillance, and their decline during chronic SIV highlights their responsiveness to neuroinflammation.

## Abstract 2

CCR7+ CD4 T cells in the brain resemble peripheral central memory cells with functional competence, and their decline during chronic SIV infection is linked to neuroinflammation.

## Graphical abstract

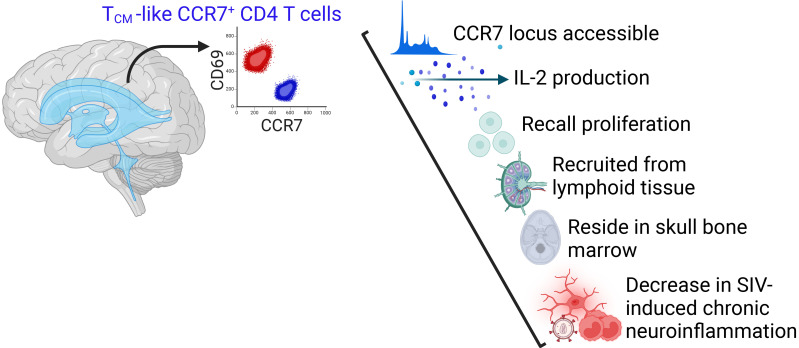

## Introduction

Antigen-experienced T lymphocyte subsets, encompassing central (T_CM_), effector (T_EM_), and tissue resident memory T cells (T_RM_), actively survey and inhabit major organ systems, contributing to immune defense and tissue function (1). Lymphocyte surveillance of the CNS primarily takes place at 2 barriers: the blood-cerebrospinal fluid barrier (BCSFB) and the blood-brain barrier (BBB). The CSF serves as a key site for T lymphocyte ingress into the CNS during homeostasis, functioning as an immunological equivalent of lymph (2). CSF CD4^+^ T cells express markers such as CCR7, CD27, and CD45RO, which are characteristic of T_CM_ cells (3, 4). Beyond the CSF, recently, CD8 T_RM_ cells expressing CD69 and CD103 have been identified in the human brain (5). While T cells reside in CNS niches during homeostasis, our understanding of specific CD4^+^ T cell subsets in the CNS and its border tissues remains incomplete. Bridging this gap is important to understand underpinnings of immune dysregulation during neuroinflammation. This is particularly relevant in HIV infection, where CD4^+^ T cells are primary targets, and chronic neuroinflammation persists in patients infected with HIV, despite virological suppression (6).

We utilized a nonhuman primate model, the rhesus macaque, to study memory CD4^+^ T cell subsets in the CNS. The rhesus macaque is particularly suited for CNS immunology research due to its close similarity to humans, including genetic diversity, a specialized neocortex, and complex meningeal structures, including extensive dural and leptomeningeal layers. These features, along with a comprehensive spectrum of memory T cell differentiation states, make it an excellent model for exploring CNS T lymphocyte function. Additionally, perfusing the macaque brain enables precise identification of local brain immune populations while minimizing vascular contamination.

Diverging from the conventional distribution patterns of memory T cells in nonlymphoid tissues, we report that distinct CD69 and CCR7 CD4^+^ T cell subsets populate the macaque brain parenchyma. CNS CCR7^+^ CD4^+^ T cells exhibit phenotypic and functional features of T_CM_ cells, including production of interleukin 2 and the capacity for rapid recall proliferation. Furthermore, CCR7^+^ CD4^+^ T cells reside in the skull bone marrow. Our findings show decreased frequencies of this subset during SIV-induced chronic neuroinflammation, emphasizing the responsiveness of CCR7^+^ CD4^+^ T cells to CNS disruptions.

## Results

### Single-cell transcriptomic analysis of CD45^+^ leukocytes identifies core T cell gene signatures in the rhesus brain.

**Figure 1:**
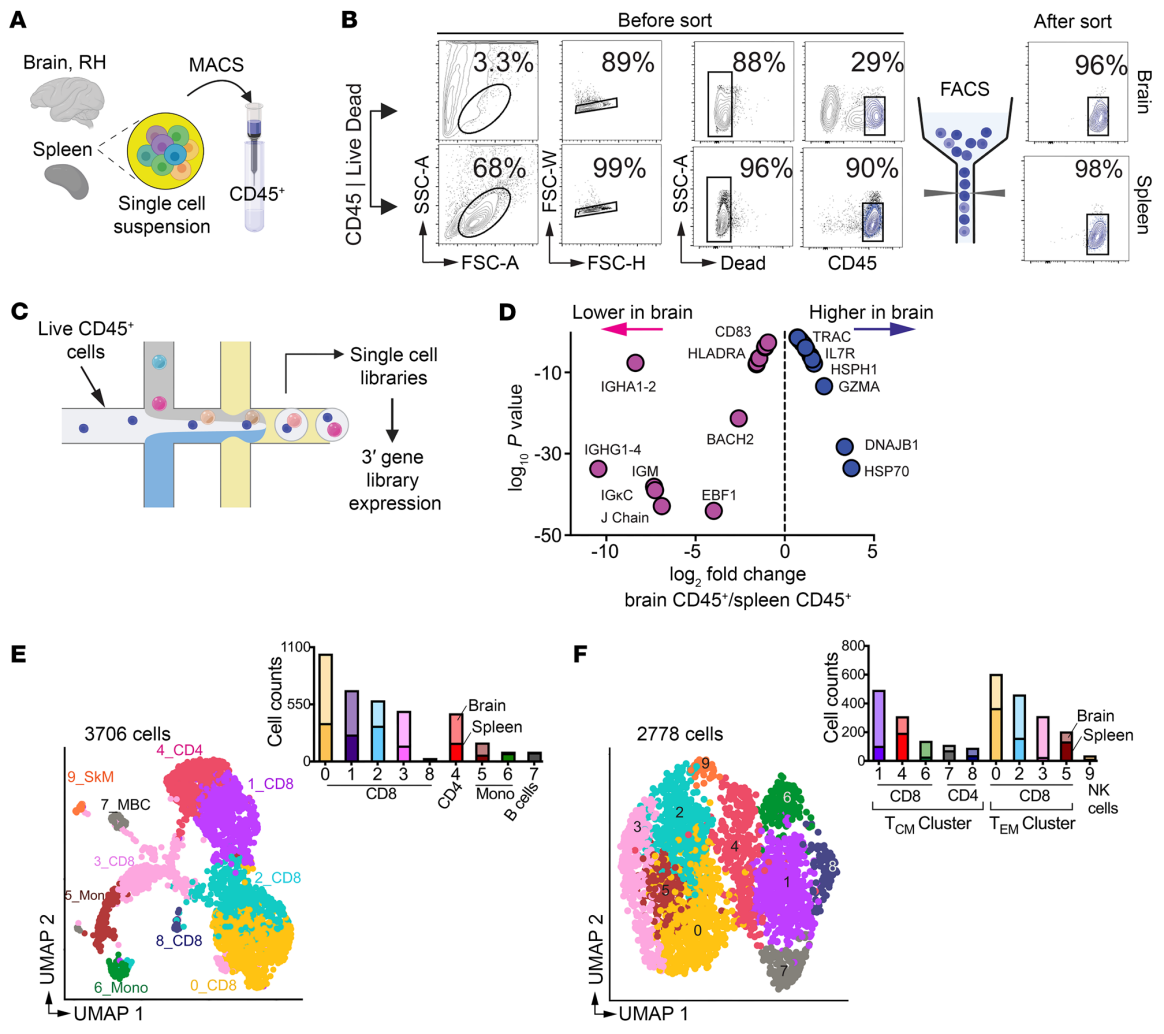
Single-cell transcriptomic analysis of CD45^+^ leukocytes identifies core T cell gene signatures in the rhesus brain. (**A**–**C**) Schematic of single CD45^+^ cell profiling in brain, right hemisphere (RH) and spleen. (**D**) Differences in B and T cell transcripts in brain versus spleen. (**E**) UMAP of scRNA-Seq transcriptional profiles from brain and spleen identifies 10 clusters. Cell clusters are color-coded based on cell identity assigned using Single R. SkM, skeletal muscle; MBC, memory B cells; Mono, monocytes. Inset shows cell proportions in each cluster split by tissue type (bottom, spleen; top, brain). (**F**) UMAP shows 10 subclusters from T cell clusters in **E**.

We previously identified T cell transcripts within synapse-dense brain regions through RNA-Seq (7). However, paucity of T cells amidst a predominance of neuronal and glial transcripts limited our assessment of T cell heterogeneity. To bridge this gap, we applied single-cell (sc) transcriptomics on cryopreserved CD45^+^ cells to elucidate transcriptional networks underlying memory T cell states in the noninflamed brain parenchyma. T cells, distinguishable by flow cytometry, constituted an average of 20% of CD45^+^ cells with a CD4:CD8 ratio of 0.2:1 (Supplemental Figure 1; supplemental material available online with this article; https://doi.org/10.1172/JCI175332DS1). scRNA-Seq was performed on viably frozen CD45^+^ cells isolated from healthy macaque brain and spleen (Figure 1A). We enriched CD45^+^ cells for sequencing by positive selection and sorting for purity and viability (Figures 1, B and C). A median of 4,952 and 3,151 CD45^+^ cells from the brain and spleen, respectively, were sequenced, resulting in 19,000 sc transcriptomes passing quality control (Supplemental Figure 2, A–E). Marker gene analysis validated our approach, demonstrating high CD45 (*PTPRC*) expression (Supplemental Figure 2F).

Transcriptome comparisons across tissues showed unique enrichment profiles. The spleen had a more pronounced B cell signature than the brain (Figure 1D), particularly in immunoglobulin-related genes (*ENSMMUG00000015202* [human orthologs IGHG1-4], *ENSMMUG00000002764* [human orthologs IGHA1 and IGHA2], *IGHM*, *IGKC*) and genes regulating B cell functions (*EBF1*, *BACH2*, *RelB*), antigen presentation (*CD74*, *HLADMB*, *HLADRA*), and signaling (*ALCAM*, *CD83*, *TRAF3*) (8). In contrast, CD45^+^ cells in the brain showed enriched T cell gene signatures. This included the T cell receptor α constant gene (*TRAC*), TCR signaling regulators (*TAOK3*, *Sos1*) (9), T cell metabolism-associated genes (*ERN1* and *TXNIP*) (10), and genes regulating effector and T_CM_ programs (*HSP70*, *DNAJB1*, *HSPH1*, *GZMA*, *ID2*, HELIOS [encoded by *IKZF2*], and *IL-7R*) (11). We verified predominance of B cells in spleen and T cells in brain through marker gene analysis and cell type cluster annotation (Supplemental Figure 2, G and H).

### Noninflamed brain harbors both effector memory and T_RM_ CD8^+^ T cells.

We next pursued high-resolution unsupervised clustering with automated label transfer using blueprint_encode (12, 13). Ten cell clusters were identified, with manual inspection and marker gene analysis (14) confirming 6 as T cells (Figure 1E and Supplemental Figure 3A). These T cell clusters were isolated and independently reclustered, identifying 3 shared T cell subtypes between brain and spleen: Terminal effector memory (T_EM_) CD8^+^ T cells (T_EM_ 8 cluster; C0, C2, C3, C5), T_CM_ CD8^+^ T cells (T_CM_ 8 cluster; C1, C4, C6) and T_CM_ CD4^+^ cells (T_CM_ 4 cluster; C7 and C8) (Figure 1F and Supplemental Figure 3B).

**Figure 2:**
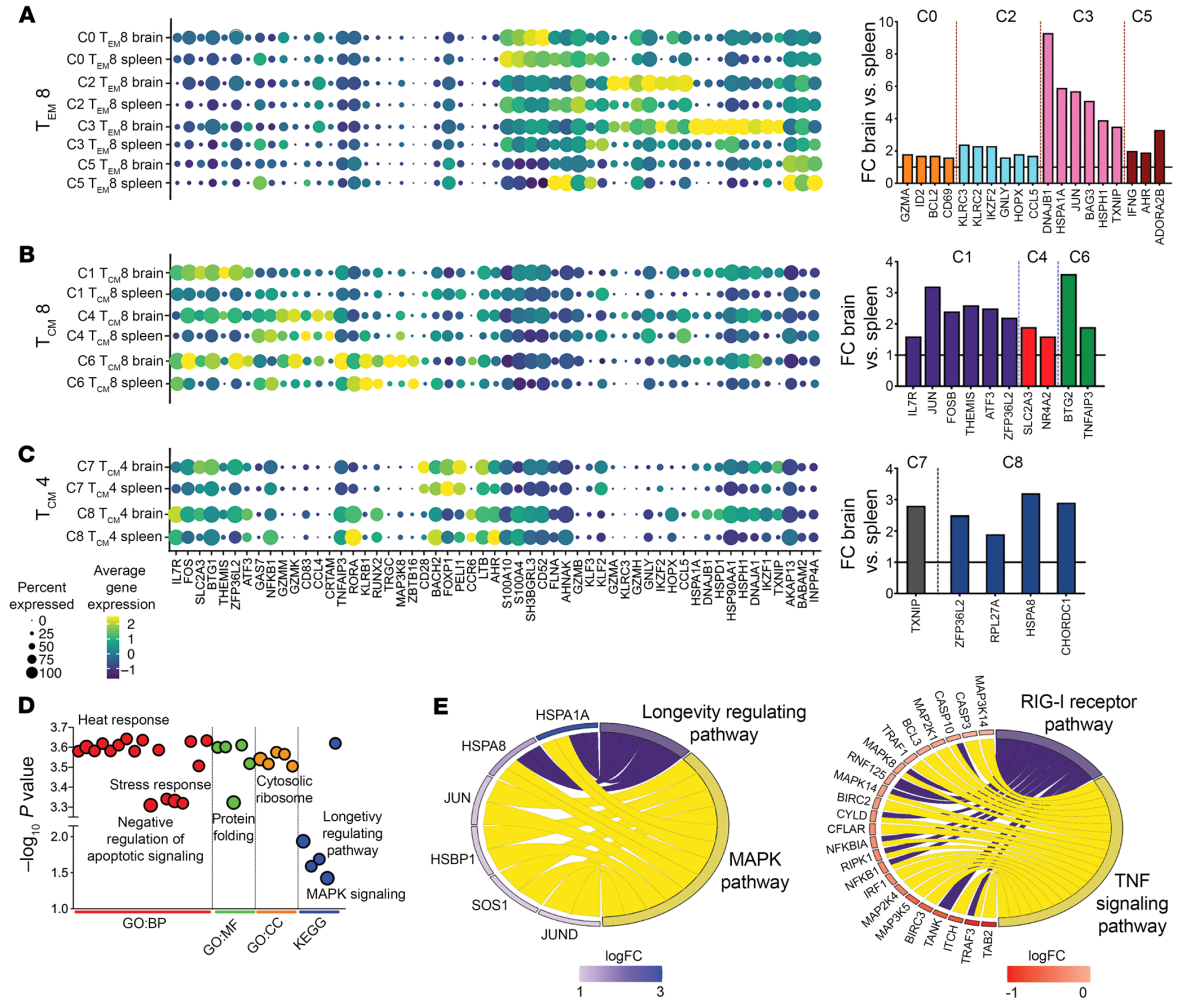
T cell clusters in rhesus brain. (**A**–**C**) Select marker genes of cell clusters. Dot size represents proportion of cells expressing a gene and color designates expression level. Bar graphs represent genes significantly higher in brain relative to spleen for indicated clusters. (**D**) Dot plot displays link between genes and pathways from GO biological processes (GO:BO), GO molecular functions (GO:MF), and GO cellular component (GO:CC) and KEGG. (**E**) Chord plots show pathways and corresponding genes enriched versus underrepresented in T_CM_4 cell clusters.

The T_EM_ 8 clusters (C0, C2, C3, C5) displayed varied gene expression (Figure 2A and Supplemental Figure 3, C and D), highlighting functional diversity in brain CD8^+^ T_EM_ cells. C0 showed genes typical of T_EM_ cells, such as S100 calcium- binding proteins (*S10010*, *S100A4*), *SH3BGRL3* expressed by T helper 1 (T_h_1) cells, regulatory receptor *CD52*, molecules driving T cell activation (*FLNA* and the scaffold protein *AHNAK*), cytolytic molecule *GZMB*, and transcription factors (TFs) *KLF2* and *KLF3*. C2 was rich in cytotoxic molecules like *GZMA*, *GZMK*, *GZMB*, *GNLY*, *KLRC3*, *HELIOS*, and *HOPX*, akin to human KIR^+^ CD8^+^ T cells (15). In contrast, C3 was enriched for DNAJ/Heatshock genes regulating memory T cell quiescence, Ikaros (encoded by *IKZF1*) and *TXNIP*, which suppress proliferation and inflammatory cytokines in T cells (16). C5 was characterized by genes linked to cell cycle progression and survival (*AKAP13*, *BABAM2*, *INPP4A*). Elevated expression of effector (*GZMA*, *KLRC2-3*, *CCL5*, *IFNG*), residency, and longevity genes (*ID2*, *AHR*, *IKZF2*, *HOPX*, *CD69*, *BCL2*) in the brain versus spleen (Bar graph in 2A) suggested that brain CD8^+^ T_EM_ cell clusters encompassed T_RM_ and T_EM_ subsets, aligning with observations in mouse and human studies (5, 17).

### Single-cell transcriptomic analysis reveals CD4^+^ and CD8^+^ T_CM_ subsets in brain.

We shifted our focus to the remaining CD8^+^ T cell clusters (C1, C4, C6) annotated as T_CM_. There were notable gene expression differences between brain and spleen (Figure 2B and Supplemental Figure 3E), with brain C1 showing higher expression of memory-related genes (*IL-7R*, *JUN*, *FOSB*, *THEMIS*), along with antiinflammatory regulators (*ATF3*, *ZFP36L2*, *NR4A2*). In addition, C1 expressed genes regulating mitochondrial function and memory cell maintenance (GLUT3, BTG1). C4 cells exhibited elevated levels of cytotoxicity and residency markers (*GAMM, GZMK, CRTAM*), and *NFκB*, with reduced *IL-7R* expression. Brain C4 also showed TCR activation markers (*SLC2A3*, *NR4A2*), suggesting potential reactivation. C6 was distinguished by an abundance of *TNFAIP3*, which inhibits IFN-γ and TNF-α in CD8^+^ T cells. These signatures of brain CD8^+^ T_CM_ clusters indicate specialized roles in memory, effector, and regulatory functions.

2 CD4^+^ T_CM_ clusters, C7 and C8, were discerned among CD4^+^ T cells. C7 was characterized by abundance of costimulatory molecules (*CD28*, *ICOS*), *IL-7R*, and the survival-related TF, *BACH2*, as well as quiescence-associated *FOXP1*, negative regulator of T cell activation, *PELI1*, memory-associated genes (*LTB*, *MAF*, *NFATC1*), and integrin *ITG-β1* (Figure 2C and Supplemental Figure 3F). C8 shared memory gene expression with C7 (*IL-7R*, *BACH2*, *LTB*), and expressed T_h_17-associated genes (*CCR6*, *AHR*, *RORA*). Gene set enrichment analysis showed alignment with longevity and MAPK pathways, and downregulation of effector pathways such as NFκB, RIG-I and TNF signaling (Figure 2, D and E). Overall, scRNA-Seq analysis of CD45^+^ cells revealed a spectrum of T cell states in the brain and spleen.

### T_CM_ and/or T_RM_ loci accessible in T cells within the brain.

**Figure 3:**
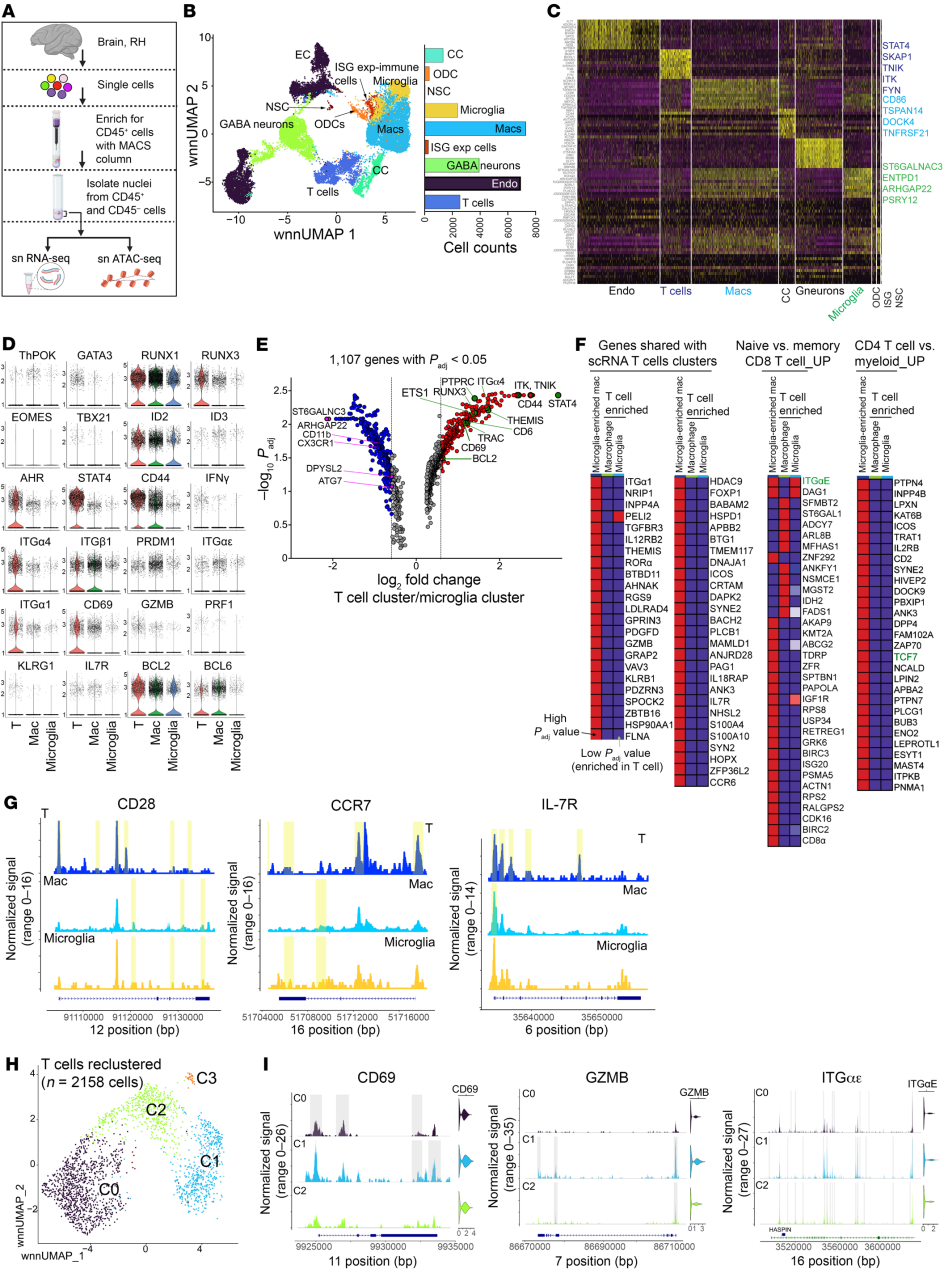
T_CM_/T_RM_ loci accessible in T cells within the brain. (**A**) Schematic of snRNA analysis. (**B**) UMAP projection of 25,321 snRNA-Seq profiles. Dots represent individual cells, and colors indicate cluster identity (labeled on right). EC, endothelial cells; NSC, neural stem cells; CC, cancer cells; Macs, macrophages; ODC, oligodendrocyte precursor cells; ISG exp cells, interferon stimulated gene expressing cells. (**C**) Heat map representation of RNA-Seq of cluster-specific marker genes across all clusters. (**D**) Violin plots show expression of key genes across immune clusters. (**E**) Gene expression differences between T cell and microglial cell clusters. (**F**) GSEA of shared genes across sn and sc analysis. (**G**) Genomic regions showing snATAC-Seq tracks of chromatin accessibility of T_CM_ genes across T cell, microglia, and macrophage immune clusters. (**H**) UMAP projection of 3 major T cell subclusters (2,158 T cells). (**I**) Genomic regions showing snATAC-Seq tracks of chromatin accessibility of T_RM/EM_ genes across 3 major T cell clusters (C0–C2) in **H**.

To explore mechanisms regulating T_CM_ and T_RM_ differentiation and validate our scRNA-Seq data, we profiled the transcriptome and epigenome in parallel. We isolated nuclei from CD45^+^ and CD45^–^ cells extracted from brain (Figure 3A) and generated over 1.5 billion reads across 47,000 nuclei, with an average of 1,378 genes/nuclei (Supplemental Figure 4, A–D). Transcriptome classification revealed distinct cell clusters, including glial cells (microglia, oligodendrocytes), neurons, endothelial cells, cancer cells, and T cells (Figure 3B and Supplemental Figure 4, E and F). The largest immune cluster was comprised of macrophages, microglia, and T cells, with each cluster expressing genes encoding proteins with known cell-type distinctions. Specifically, cells in the macrophage cluster expressed *CD86*, *TSPAN14*, and *TNFRSF21*. The microglia cluster expressed *ST6GALNAC3*, *ENTPD1*, and *P2RY12*, while T cell clusters expressed the T_h_1 TF *STAT4*, T cell adaptor protein *SKAP1*, as well as kinases and signaling molecules *TNIK*, *ITK*, and *FYN* (Figure 3C and Supplemental Figure 4F).

We identified expression of several key genes regulating T cell differentiation and function, including zinc finger TFs (*ThPOK*, *GATA3*), Runx TFs (*RUNX1* and *RUNX3*), T-box TFs (*EOMES*, *TBX21*), inhibitor of DNA binding proteins (*ID2*, *ID3*), TFs regulating cytokine production (*AHR*, *STAT4*), markers of antigen-experienced cells (*CD44*, *IFNG*, *ITG-α4*, *ITG-β1*), markers of T cell residency (*PRDM1*, *ITG-α1*, *ITG-αE*, *CD69*, *GZMB*, *KLRG1*, *PRF1*), and markers of long-lived cells with T_CM_ features (*BCL2*, *BCL6*) (Figure 3D).

To quantitatively evaluate genes enriched in T cells, we conducted differential gene expression (DGE) analysis across T cell and microglial clusters. Within microglia, we discovered enrichment of canonical brain resident microglia transcripts, including *CX3CR1*, *ITG-αM*, *TMEM*, and *SIGLEC* (18–20) (Figure 3E). In contrast, genes highly expressed by the T cell cluster included those regulating T cell signaling (*TRAC*, *ITK*, *THEMIS*, *TNIK*), TFs controlling CD4 and CD8 T cell programs (*STAT4*, *RUNX3*), T cell migration (*CD44*, *ITGα4*), residency (*CD69*), and T cell survival (*BCLA11B*, *BCL2*), including the TF *ETS-1*, which regulates IL-7R expression (21) (Figure 3E and Supplemental Figure 4G). Genes involved in T_h_17 function, *ROR-α*and *AHR* were also expressed in keeping with the transcriptome of T_CM_4 C8. Further, a similar T cell gene expression profile was observed when comparing macrophages to T cells, with the IL-12–induced CD4 T_h_1 TF, *STAT4* being the most highly expressed gene in T cells (Supplemental Figure 4H). ATAC-Seq analysis highlighted open chromatin in regulatory regions of STAT4, especially within the T cell cluster, aligning with high STAT4 expression (Supplemental Figure 4I). Additionally, downstream targets of *STAT4*, including *IFN-γ*and *ICOS*, were distinctly expressed in T cells, differing from patterns in innate immune cells (Supplemental Figure 4J). These genes also featured as top markers in the T cell cluster in our scRNA-Seq of CD45^+^ cells.

To formally ascertain whether T cell clusters expressed genes overlapping with our scRNA-Seq profiles, we examined 7,798 transcripts from single nucleotide RNA-Seq–derived (snRNA-Seq–derived) immune clusters. We then used DGE *P* values for each expressed gene in T cells relative to macrophages and microglia. For comparison, we also included DGE *P* values of microglial transcripts relative to macrophages. Gene set enrichment analysis showed overlap with top 20 marker genes expressed by scRNA-Seq T cell clusters, including classical effector/memory transcripts such as *GZMB*, *CRTAM*, and *IL-7R*, among others (Figure 3F). Additionally, when aligning these DGE genes with known T cell signatures from mouse studies, we found that ITG-αE (integrin receptor for T_RM_) and TCF7 (TF critical for T_CM_ development) were notably present in T cells over macrophages and/or microglia (*P*_adj_ < 0.05).

To assess T_CM_ gene accessibility, we focussed on genes vital for T_CM_ function and survival — *CD28*, *IL-7R*, and *BCL2* — which macrophages and microglia also expressed. T cells exhibited increased ATAC peaks for *CD28* and *IL-7R*, suggesting an open chromatin configuration, especially in their promoter regions (Figure 3G), whereas *BCL2* accessibility was similar across immune cells (Supplemental Figure 4K). Despite low expression, *CCR7* chromatin accessibility was higher in T cells, contrasting with CCR7 absence in innate immune cells. Additionally, our analysis of gene expression patterns and motif enrichment, using the HOMER database, revealed that TFs from the bZIP, RUNT, and ATF families, pivotal in regulating T_CM_ genes, were significantly enriched, marking about 30% of target sequences in T cells.

To pinpoint genes controlling memory T cells states, we reclustered 2,158 T cells, which revealed 4 distinct clusters (Figure 3H). Since CCR7 was expressed in less than 1% of cells across all clusters, we probed the promoter accessibility within T_RM_ genes across 3 major T cell clusters (C0–C2). With *CD69* and *GZMB* marking T_RMs_ and their expression in C1, we anticipated and confirmed ATAC peaks for key T_RM_ genes in C1. Indeed, peak tracks showed increased chromatin accessibility for regulatory regions of *CD69*, *GZMB*, and *ITG-αE* in C1 (Figure 3I). The sequencing outcomes indicate that the primate brain harbors T cells with diverse chromatin accessibility landscapes for genes that govern residency and migration, suggesting the presence of T cells with potential resident and central memory features.

### CCR7^+^ CD4^+^ T cells in CNS share phenotypic features with T_CM_ in blood and lymph nodes.

To validate and extend our sequencing observations, we investigated the immune makeup of the CSF. CSF samples were collected from the foramen magnum, alongside paired axillary lymph node aspirates and blood samples (Supplemental Figure 5A). Unlike blood, CSF exhibited minimal B cell and monocyte presence and a preferential infiltration of T cells at steady state.

CSF T cells showed predominance of CD28^hi^ CD4^+^ memory T cells, with an absence of terminally differentiated (CD28^–^ CD95^+^) and naive (CD28^int^ CD95^–^) subsets, consistent with human phenotypes (22). Relative to CD28^hi^ subset in blood, approximately 50% of CSF CD28^hi^ CD4^+^ T cells expressed CCR7, compared with 20% in CD28^hi^ CD8^+^ T cells (Supplemental Figure 5, B and C).

**Figure 4:**
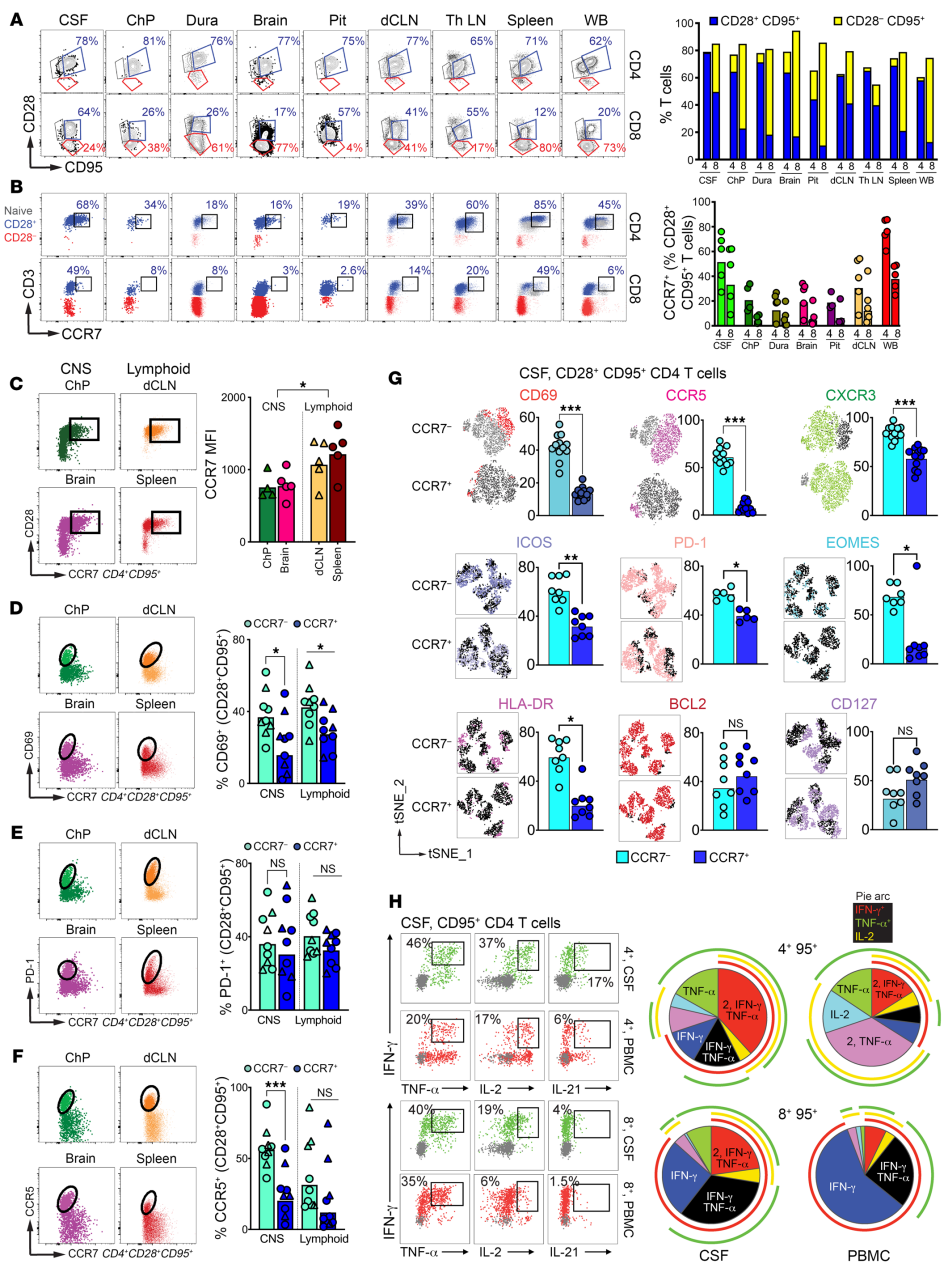
CCR7^+^ CD4^+^ T cells in CNS share phenotypic features with T_CM_ in blood and lymph nodes. (**A**) Representative flow plots illustrate CD28 and CD95 expression on CD4^+^ and CD8^+^ T cells; frequencies of CD28^+^ CD95^+^ (blue) and CD28^–^ CD95^+^ (yellow) in CD4^+^ T cells and CD8^+^ T cells. (**B**) Representative flow plots illustrate CCR7 expression on CD28^Hi^ CD4^+^ (top row) and CD28^Hi^ CD8^+^ (bottom row) T cells; frequencies of CCR7 expression on CD28^Hi^ CD4^+^ and CD28^Hi^ CD8^+^ T cells. (**C**) Representative flow plots illustrate CD28 expression and CCR7 expression on CD4^+^ CD95^+^ T cells in CNS and lymphoid tissues; CCR7 MFI of CD4^+^ CD95^+^. (**D**–**F**) Representative flow plots indicating CD69, PD-1, CCR5, and CCR7 expression on CD4^+^ CD28^+^ CD95^+^ T cells in CNS and lymphoid tissues; frequency of CD69^+^, PD-1^+^, and CCR5^+^ on CD4^+^ CD28^+^ CD95^+^ CCR7^–/+^ T cells. (**G**) Representative tSNE plot illustrating expression of T cell markers on CD4^+^ CD28^+^ CD95^+^ CCR7^–/+^ T cells in the CSF; frequencies for each population. (**H**) Representative flow plots illustrating cytokine production in the CSF and PBMCs. CSF, cerebrospinal fluid; ChP, choroid plexus; Pit, pituitary; dCLN, deep cervical lymph nodes; Th LN, thoracic lymph node. WB, whole blood; PBMC, peripheral blood mononuclear cells. *P < 0.05, **P < 0.01, ***P < 0.001.

We compared phenotypes of CSF-derived T cells to those in matched CNS tissues and adjacent lymph nodes, spleen, and blood. Brain T cells showed distinct differentiation states: CD4^+^ T cells were enriched for CD28, while CD8^+^ T cells were mostly CD28^–^ (Figure 4A). As in the CSF, CD28^hi^ CD4^+^ T cells in the parenchyma showed varied CCR7 expression (Figures 4B). We assessed CCR7^+^ CD4^+^ T cells in the CNS (choroid plexus and brain parenchyma) to those in corresponding lymphoid compartments (deep cervical lymph nodes and spleen) to identify similarities to T_CM_. Analysis of receptor expression revealed lower per cell CCR7 expression in the CNS than in lymphoid tissues. (Figure 4C).

CCR7^+^ CD4^+^ T cells in each tissue were less likely to express CD69, a key T_RM_ marker (Figure 4D). PD-1 levels, indicative of TCR stimulation, were similar across subsets, aligning with findings in CD8^+^ T_RM_ (23) (Figure 4E). Given known CCR5 expression in intestinal CD69^+^ CD4^+^ T_RM_ (24), we explored CCR7^–^ CD4^+^ T cells for increased CCR5 alongside higher CD69 levels. This pattern was confirmed in the CNS, but not in lymphoid tissues (Figure 4F). Thus, CD4^+^ T cells within the noninflamed brain parenchyma mainly show CD28^hi^ expression, bifurcating into subsets based on CD69 and CCR7, with CCR7^+^ T cells resembling their lymphoid counterparts.

We performed FLOW SOM clustering on CSF CD4^+^ T cells (*n* = 4) to determine if CCR7^+^ and CCR7^–^ subsets represent distinct clusters. Based on expression of specific T_h_1 (CXCR3, CCR5), T_h_17 (CCR6), activation, and memory markers (CD95, PD-1, CD69), 5 metaclusters were defined with varying levels of CCR7 expression (Supplemental Figure 6A). CCR7^–^ and CCR7^+^ clusters showed enrichment of distinct surface markers; CCR5 and PD-1 were enriched in the CCR7^–^ cluster, while CCR6 was enriched in the CCR7^+^ cluster (Supplemental Figure 6B).

We then examined if CCR7^+^ CD4^+^ T cells in the CSF resembled quiescent T_CM_, and conversely, whether CCR7^–^ CD4^+^ T cells exhibited T_RM_ or activated T_EM_ cell features. Unlike blood CCR7^–^ CD4^+^ T cells in CSF predominantly expressed CD69, reflecting patterns in the brain (Figure 4G and Supplemental Figure 7). CSF CCR7^–^ subset also showed higher expression of CCR5 and CXCR3, as well as activation markers like ICOS, EOMES, PD-1, and HLA-DR. Both subsets expressed CD127 and BCL-2. In summary, CCR7^+^ CD4^+^ T cells in the CSF and brain display core T_CM_ traits, like those in lymphoid tissues. We postulated that these CNS CD4^+^ T cells would exhibit functional hallmarks of T_CM_ by producing IL-2. Upon PMA/Ionomycin stimulation, CSF CD4^+^ T cells indeed demonstrated polyfunctionality, including IL-2 production (Figure 4H).

### Sequestration of CD4^+^ T_CM_ in lymphoid tissues reduces CCR7^+^ CD4^+^ T cell frequencies in CSF and increases soluble inflammatory markers.

**Figure 5:**
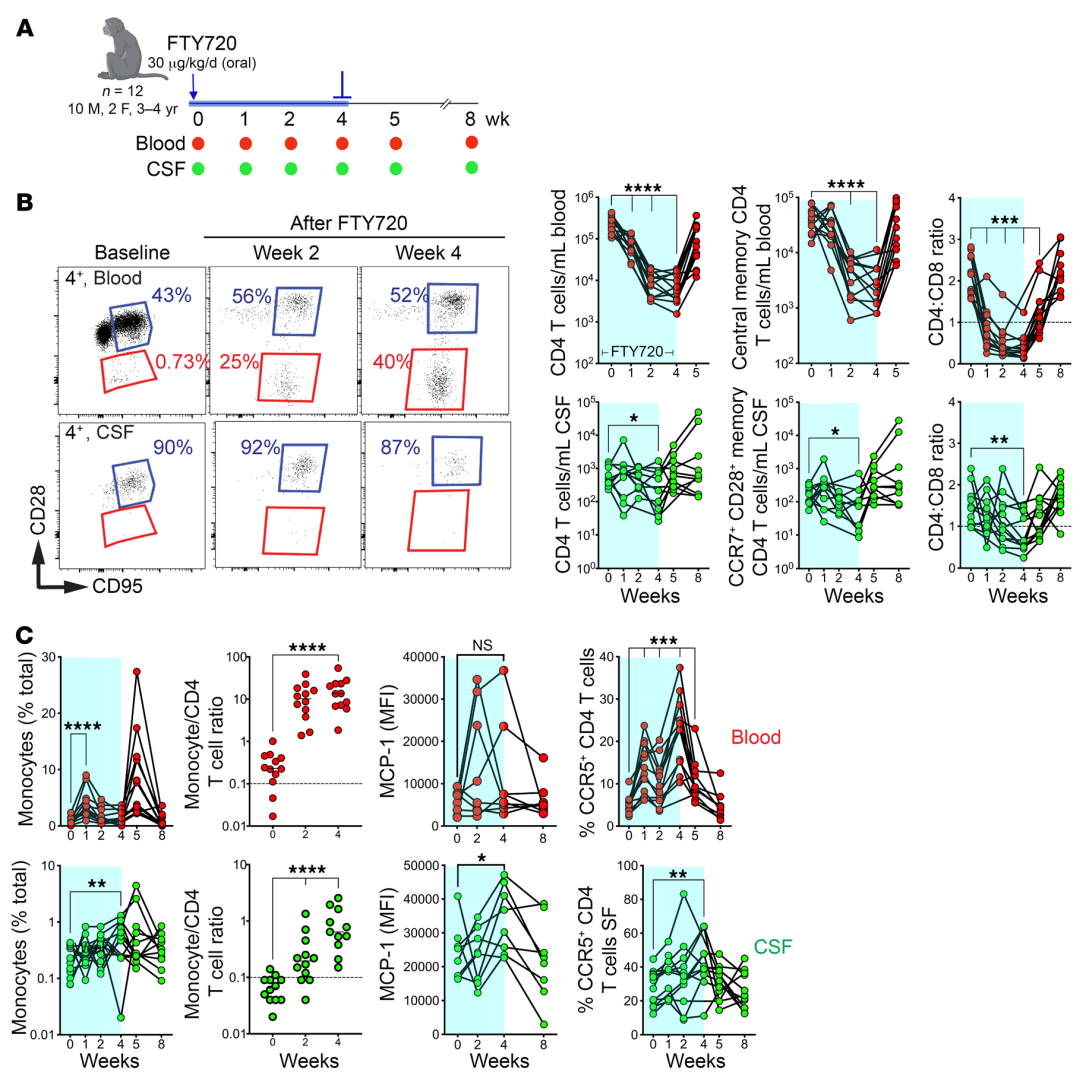
Sequestration of CD4^+^ T_CM_ in lymphoid tissues reduces CCR7^+^ CD4^+^ T cell frequencies in CSF. (**A**) Study schematic: *n* = 12 rhesus macaques (ages 3–4 years) were administered an oral dose of 30 μg/kg per day of FTY720 for the first 4 weeks of the study. CSF taps and blood draws were performed at indicated time points. (**B**) Representative flow plots indicating CD28 and CD95 expression on CD4^+^ T cells from the blood (top row) or the CSF (bottom row) (Left); CD4^+^ T cell counts/mL, CD4^+^ T_CM_ cells and CCR7^+^ CD28^+^ memory CD4^+^ T cells/mL blood or CSF, and CD4-to-CD8 ratio for blood and CSF (Right). (**C**) Frequencies of monocytes, monocyte-to-CD4^+^ T cell ratio, median fluorescent intensity (MFI) of monocyte chemoattractant protein-1 (MCP-1), and CCR5 expression of CD4^+^ T cells in the blood and CSF over the course of the study. *P < 0.05, **P < 0.01, ***P < 0.001, ****P < 0.0001.

To determine if CNS CCR7^+^ CD4^+^ T cells displayed migration patterns to and from lymphoid tissue, a hallmark of T_CM_, we explored the effect of FTY720, known to trap T_CM_ cells in lymph nodes, on T_CM_ frequencies in the CSF. We treated 12 rhesus macaques with FTY720 (30 μg/kg/day) for a month (Figure 5A) and analyzed paired blood and CSF T cells over 8 weeks, tracking T cells within the subarachnoid space (SAS) through FTY720-induced lymphocyte depletion and the subsequent rebound posttreatment.

Analysis of blood T cells showed rapid decline in total CD4^+^ T cells a week after FTY720, while CD4^+^ T cell counts significantly decreased in CSF at week 4 after FTY720 (Figure 5B). Consistent with FTY720-mediated inhibition of S1PR-mediated T cell egress and retention of CCR7^+^ T cells in lymph nodes in macaques (25), rapid sequestration of naive T cells, the subset exhibiting the highest per-cell expression of CCR7, ensued. This was evidenced by a notable 4-fold reduction in the absolute counts of naive (CD28^+^ CD95^–^) T cells within week 1 of FTY720.

By week 4, there was an additional reduction in naive T cells, marked by a 100-fold decrease in CD4^+^ T cells and an 80-fold decline in CD8^+^ T cells compared with baseline (Supplemental Figure 8A). The heightened CD4^+^ T cell decline can be attributed to their shorter lymph node residency time, rendering them more susceptible to mechanisms impeding their egress (26). It took up to 2 weeks for a significant decrease in the peripheral CD4^+^ T_CM_ pool to manifest, culminating in a 17-fold reduction by week 4. This decline was accompanied by a significant decrease in CSF CCR7^+^ CD4^+^ T cells by week 4, indicating recruitment of CCR7^+^ CD4^+^ T cells into the SAS from lymphoid tissues via the systemic compartment.

While blood CD8^+^ T_CM_ frequencies fell (Supplemental Figure 8B), CSF CD8^+^ T cells remained stable, potentially due to CD28^–^ CD95^+^ CD8^+^ T cells in the CSF, which are mostly CCR7^–^ and are less affected by FTY720. This could also reflect their migration from nonlymphoid tissues. Consequently, the CSF CD4-to-CD8 ratio significantly dropped by week 4. The blood showed an increased frequency of CD28^–^ T_EM_ early on, with CD4^+^ and CD8^+^ T cells CM-to-EM ratios shifting at weeks 1 and 4, consistent with the expected effect of CCR7^–^ EM cells not being retained in lymph nodes. However, the CSF T cell CM-to-EM ratio remained unchanged throughout the 4 weeks, indicating a tight regulation that limits CD28^–^ CD95^+^ CD4^+^ T cells from entering the SAS (Figure 5B). Cytokine analysis showed a transient decrease in T_h_1, T_h_17, and regulatory cytokines in plasma after treatment, demonstrating extensive effects on CD4 helper subsets, although these cytokines were undetectable in the CSF (Supplemental Figure 8C).

In mice, T cell depletion from meninges induces proinflammatory innate immune skewing (27). We therefore examined monocyte frequencies after FTY720 to gauge compensatory increase in the SAS. The data showed a net increase in monocytes in the CSF at week 4, significantly increasing the monocyte-to-CD4^+^ T cell ratio (Figure 5C). Significant elevation of CSF, but not blood, monocyte chemotactic protein-1 (MCP-1) at week 4 implied CSF monocyte influx was chemokine mediated. Moreover, CSF CCR5^+^ CD4^+^ T cells significantly increased, and there was an inverse association (r = –0.73; *P* < 0.01) between CCR7^+^ and CCR5^+^ CD4 T cells (Supplemental Figure 8D). In conclusion, the data suggest that CSF CCR7^+^ CD69^–^ CD4^+^ T cells are mainly recruited from lymphoid tissues. Moreover, immune activation linked to reduced CCR7^+^ CD4^+^ T cells in the SAS suggests a role for these cells in neuroimmune homeostasis.

### CCR7^+^ CD4^+^ T cells in CNS exhibit functional T_CM_ features and reside within skull bone marrow.

**Figure 6:**
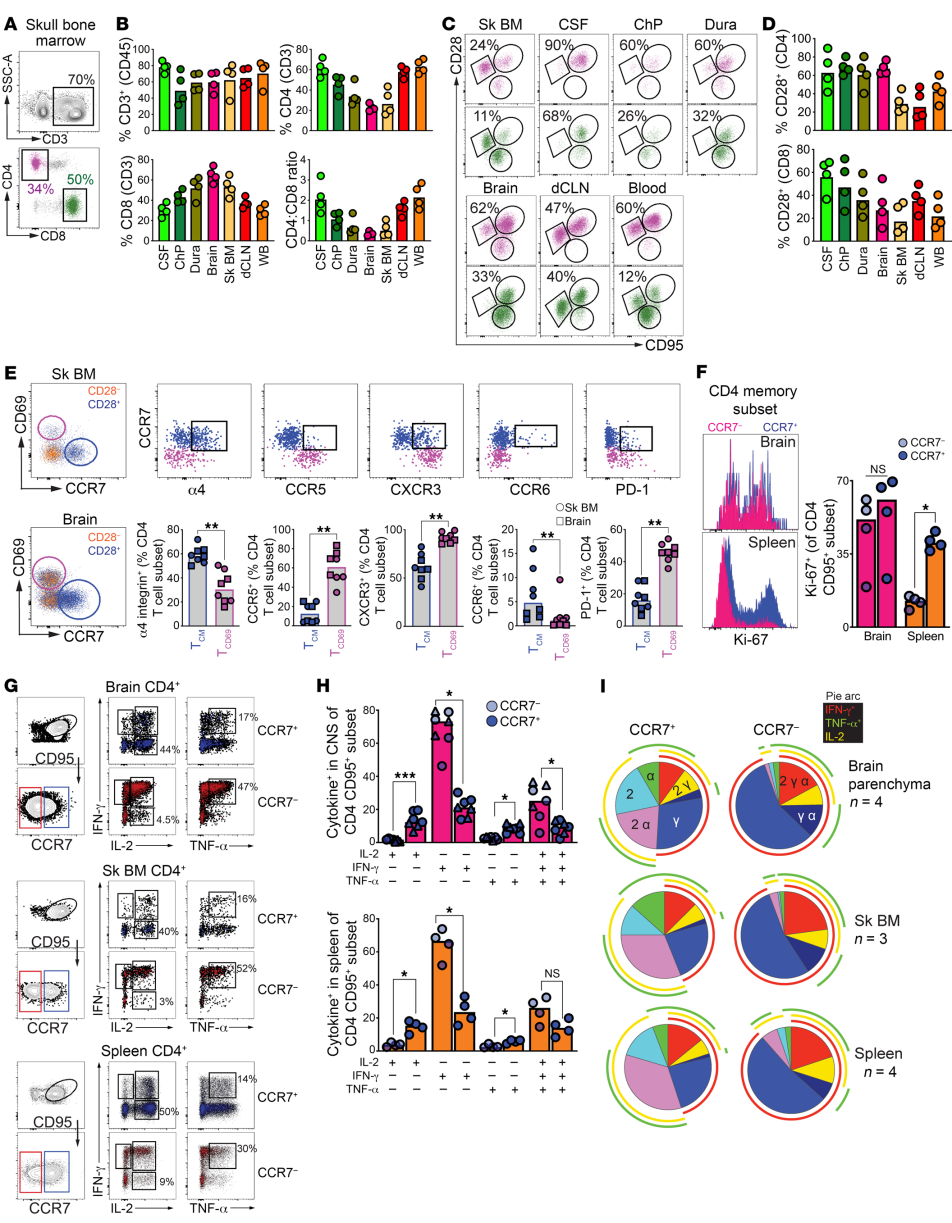
CCR7^+^ CD4^+^ T cells in CNS exhibit functional T_CM_ features and reside within skull BM. (**A**) Representative gating for T cells within the skull BM and (**B**) corresponding frequencies of CD3^+^, CD4^+^ (top), CD8^+^ T cells, and CD4-to-CD8 ratios (bottom) across tissue compartments. (**C**) Population gates for CD4^+^ (purple) and CD8^+^ (green) subsets with (**D**) corresponding frequencies of CD28^+^ subsets across tissue compartments. (**E**) Phenotypic characterization of T_CM_-like (CCR7^+^; blue) and CD4^+^ T_RM_ (CD69^+^; purple) cells from brain and skull BM. (**F**) Ki67 MFI and frequencies on CCR7^–^ and CCR7^+^ CD4^+^ T cells after T cell activation using anti-CD3 and anti-CD28. (**G**–**I**) Representative gating for CD95^+^ CCR7^+^ CD4^+^ T cells and CD95^+^ CCR7^–^CD4^+^ T cells and bar charts illustrating cytokine production after stimulating with PMA/Ionomyocin in Brain, Skull BM, and Spleen. (**I**) Pie Charts indicating cytokine functionality after PMA/Ionomycin treatment. (**A**–**F**) Data points indicate individual tissue samples. (**F**) Symbols indicate skull BM (circle) or brain tissue (square) samples. Bars indicate medians. *P < 0.05, **P < 0.01, ***P < 0.001.

Recognizing the importance of bone marrow (BM), particularly within the skull (28, 29), for T_CM_ localization we explored presence of CD4^+^ T cells with T_CM_ attributes in this niche. After manually extracting BM cells from the skull, sc suspensions were stained to identify innate and adaptive immune cells. Like mouse brain findings, our analysis identified 3 immune subsets among CD3^–^ CD45^+^ cells based on CD11b and HLA-DR expression (Supplemental Figure 9). T cells comprised 71% of the CD45^+^ population in skull BM, echoing CSF T cell prevalence (Figure 6A), with a CD8^+^-to-CD4^+^ T cell ratio mirroring that in brain tissue (Figure 6B). Like in the CNS, CCR7 expression in CD28^+^ CD4^+^ T cells was variable, while CD28^–^ CD4^+^ T cells largely lacked CCR7 (Figure 6, C and D and Supplemental Figure 10).

Phenotypic analysis revealed distinct profiles between CNS CD4^+^ T cell subsets; CCR7^+^ cells showed higher integrin-α4 and CCR6 but lower CCR5 and CXCR3 expression compared with CD69^+^ cells, which had more pronounced PD-1 expression, in both skull BM and in the brain (Figure 6E). Functional characterization of memory CCR7^–^ and CCR7^+^ CD4^+^ subsets demonstrated that both brain CCR7^–^ and CCR7^+^ CD4^+^ T cells mounted recall proliferation ex vivo, while splenic CCR7^+^ CD4^+^ T cells, and to a lesser extent CCR7^–^ CD4^+^ T cells were Ki67^+^ (Figure 6F). Analysis of cytokine production following PMA/I stimulation showed that CNS CCR7^+^ CD4^+^ T cells produced a higher relative IL-2, while the CCR7^–^ subset produced high levels of IL-2, IFN-γ, and TNF-α. This cytokine expression pattern was consistent with T_CM_ functionality, as exemplified by a similar cytokine pattern in splenic CD4^+^ T_CM_ (Figure 6, G–I). In summary, the data highlight the presence of a CCR7^+^ CD4^+^ population in the brain and skull BM exhibiting T_CM_-like characteristics, akin to T_CM_ in the spleen.

### vRNA within frontal and temporal lobes during chronic SIV infection.

**Figure 7:**
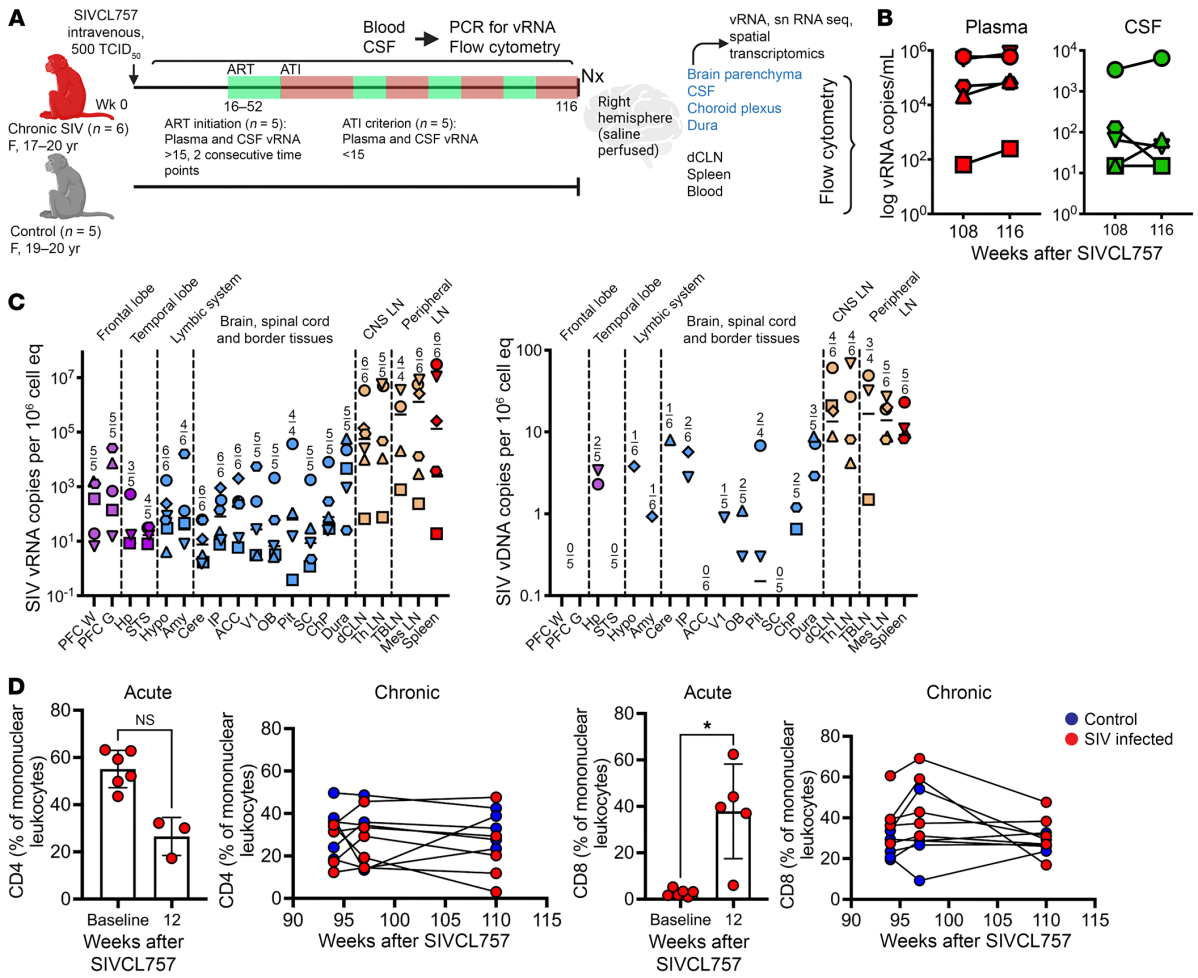
vRNA within frontal and temporal lobes during chronic SIV infection. (**A**) Study schematic: rhesus macaques were infected with SIVCL757 intravenously and longitudinally assessed for systemic and CNS viral burden, snRNA-Seq, spatial transcriptomics, and immune responses by flow cytometry. (**B**) Kinetics of plasma (red) and CSF (green) viral loads during the chronic phase (week 108–116) of SIVCL757. (**C**) vRNA and vDNA in various brain regions, dura mater, deep cervical lymph nodes, and PBMCs. (**D**) CSF CD4 and CD8 frequencies during the acute phase (week 12) and chronic phase (week 92–110) of SIVCL757 infection. PFC W, prefrontal cortex white matter; PFC G, PFC gray matter; Hp; hippocampus; STS, superior temporal sulcus; Hypo, Hypothalamus; Amy, Amygdala; Cere, Cerebellum; IP, inferior/intra parietal; ACC, anterior cingulate cortex; V1, primary visual cortex; OB, olfactory bulb; Pit, pituitary; SC, spinal cord (near base of skull); ChP, choroid plexus; dCLN, deep cervical lymph node; Th LN, thoracic lymph node; TBLN, tracheobronchial lymph nodes; Mes LN, mesenteric lymph nodes. *P < 0.05.

To better understand CD4^+^ T_CM_ in the CNS, we investigated these cells in a model of chronic viral neuroinflammation (30). Aged rhesus macaques (17–20 years) were infected with neuropathogenic SIVCL757 to elicit chronic neuroinflammation and establish CNS virus presence. After the postacute phase, to ensure CNS viral dissemination, suboptimal antiretroviral therapy (ART) was initiated during weeks 16–52 after SIV when CSF and plasma viral RNA (vRNA) levels exceeded the threshold of detection (over 15 vRNA copies/mL). This treatment was interrupted when vRNA levels fell below the threshold of detection (Figure 7A). We adopted this regimen to induce cycles of viral suppression and rebound within the CNS, simulating scenarios in individuals at risk for neurological comorbidities due to chronic neuroinflammation (6). An exception to this protocol was followed in the case of animal 33191, who did not receive ART. This decision was made because CSF vRNA levels exceeded 15 copies/mL only at a single time point during week 6 and subsequently dropped below the threshold of detection. Longitudinal collections of CSF and matched blood from infected animals were conducted for up to 116 weeks, except for 1 SIV^+^ animal (34974) that was euthanized at 52 weeks due to health complications. Before necropsy, ART was interrupted in all animals (except ART-naive 33191) to induce viral rebound. At necropsy, CNS and peripheral lymphoid tissues were collected for analysis. Age-matched control group of SIV-unexposed animals (*n* = 5) was also assessed.

Following infection, viremic animals (*n* = 4) exhibited median plasma viral loads of 165,000 copies/mL at week 3, with CSF vRNA reaching a median of 19,750 vRNA copies/mL (Supplemental Figure 11). Plasma and CSF vRNA exhibited a lower magnitude and variable pattern when compared with viral loads observed following SIVmac251 (31). Like SIVmac251, there was plasma-CSF concordance during acute SIVCL757 infection before ART initiation. An exception was observed in a TRIM5α-restrictive animal (32967), which displayed transient plasma viral discordance up to week 6 after infection. Of note, 2 animals (33191 and 34996) demonstrated sporadic and minimal vRNA in CSF, despite plasma vRNA after the acute phase. ART initiation between 16–46 weeks after infection led to viral suppression (vRNA copies < 15) in plasma as early as 4 weeks and as late as 6 weeks after ART initiation. Throughout chronic infection, viral loads in CSF were consistently 3 log-fold lower than those in plasma (median viral loads/mL at week 108, plasma, 50,000; CSF, 65), aligning with our findings in acute SHIV.C.CH505 (Figure 7B) (25).

At necropsy, 3 mm postmortem punch biopsies were collected to assess vRNA and vDNA in various brain regions, border tissues, CNS-draining lymph nodes, and peripheral lymphoid tissues (Figure 7C). The frontal lobe, linked to cognition, displayed vRNA positivity in both gray and white matter across all animals tested. However, vDNA was undetectable. The detection of vRNA and vDNA exhibited variability in the temporal lobe, limbic system, and other brain and border tissues. While the CNS-draining lymph nodes and peripheral lymphoid tissues showed vRNA in all animals, vDNA was not consistently detected across CNS tissues in certain animals. The presence of widespread vRNA within the CNS coupled with low levels of vDNA, may be attributed to ineffective viral integration of SIVCL757 within CNS myeloid and CD4^+^ T cells.

CSF lymphocyte analysis showed trend for CD4^+^ T cell reduction during the first 12 weeks of infection (not significant), while CD8^+^ T cells exhibited an increase (*P* < 0.05; fold change, 13). Both CD4^+^ and CD8^+^ T cell frequencies stabilized during the chronic phase (Figure 7D). These findings highlight widespread vRNA in the CNS, low vDNA levels, and acute changes in CD4^+^ and CD8^+^ T lymphocyte populations within the CSF following SIVCL757 infection.

### Spatial profiling of the hippocampus shows induction of neuroinflammatory and neurodegenerative gene programs during chronic SIV infection.

To assess the extent of neuroinflammatory responses during chronic SIV infection, we utilized 2 complementary methods: spatial transcriptomics on the hippocampus and sc analysis of CD45-enriched cells derived from brain parenchyma. Initially, we examined T cell distribution in the human brain by performing IHC analysis on hippocampal sections from both glioblastoma patients (GBM-01) and individuals who were nondemented from the Netherlands Brain Bank. We aimed to identify neurons (NeuN), myeloid cells (CD11b, IBA1), and lymphocytes (CD45, CD3, CD4). Healthy tonsil sections showed abundant T cells and myeloid cells and lacked neuron-specific staining. In contrast, hippocampal sections from patients who were nondemented displayed microglia, neurons, T cells, and monocytes, primarily around blood vessels (Supplemental Figure 12). Hippocampal tissue derived from patients with glioblastoma exhibited a pronounced distribution of T cells throughout the brain parenchyma.

**Figure 8:**
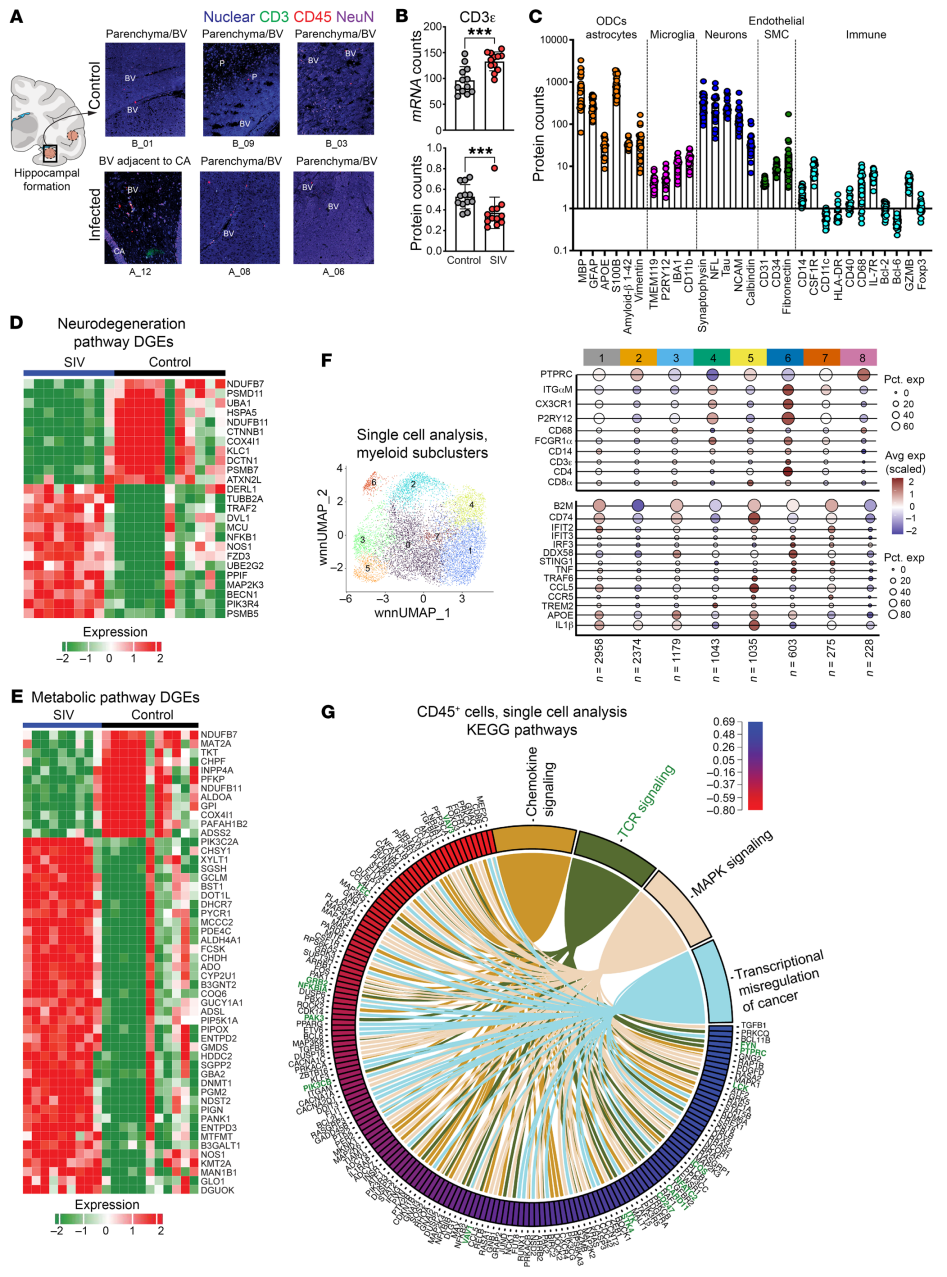
Induction of neuroinflammatory and neurodegenerative gene programs during chronic SIV infection. (**A**) Representative illustration for ROI selection within the hippocampal region of control (top) and SIVCL757-infected (bottom) animals; Nuclear (blue), CD3 (green), CD45 (red), and NeuN (purple) for Nanostring whole transcriptome analysis (WTA) and proteomics pipeline. (**B**) CD3ε mRNA and protein counts for ROIs. (**C**) Protein counts for all ROIs. (**D**) Differentially expressed neurodegenerative genes across control and SIV-infected ROIs. (**E**) Differentially expressed metabolic genes across control and SIV-infected ROIs. (**F**) UMAP plot shows cell annotation for myeloid specific gene clusters from sc data. Dot plots depict average gene expression of canonical microglia, monocyte, macrophage, antiviral, and inflammatory response genes across 8 distinct myeloid clusters. (**G**) Chord plot of differentially expressed genes across control and SIV-infected CD45-enriched cells from sc transcriptomics. Genes related to TCR signaling pathway are colored in green for clarity. ***P < 0.001.

With the presence of T cells in the human brain established by IHC, we analyzed hippocampal tissue from chronically SIV-infected macaques (1 healthy control, 33980, and 1 SIV^+^ animal, 35595) using the Nanostring Digital Spatial Profiler (DSP) platform. Using CD3, CD45, and NeuN as morphological markers to identify T cells, leukocytes, and neurons, we selected 24 regions of interest (ROIs) with varying CD45 expression levels, covering distinct spatial zones within the hippocampus. These zones included areas around CA1, small and large blood vessels, and parenchymal regions (Figure 8A). The expression of CD45, CD3, and NeuN proteins showed heterogeneity across the selected 24 ROIs. Using the fluorescence signal of CD3 and CD45, we identified T cells primarily within blood vessels. Subsequently, these 24 ROIs underwent comprehensive 147-plex antibody profiling and whole transcriptomic analysis (WTA). The data showed higher *CD3E* RNA counts and lower CD3Ε protein counts in SIV-infected ROIs, suggestive of possible CD3 protein internalization due to activation (Figure 8B). Antibody profiling revealed the expected enrichment of signals corresponding to glial cells (oligodendrocytes [myelin basic protein], astrocytes [GFAP, APOE, S100B, amyloid β, Vimentin], microglia [TMEM119, CD11b, IBA1, P2RY12]) neuronal proteins (synaptophysin, neurofilament light chain, Tau, NCAM [CD56], Calbindin), and endothelial and muscle cells (CD31, CD34, Fibronectin). With respect to immune proteins, we detected myeloid cell markers (CD14, CSF1R, CD11c, HLA-DR, CD40, CD68), memory T_CM_ markers (CD127, BCL-2, BCL-6), effector/resident cell marker (GZMB), and transcriptional regulators (BCL-6, FOXP3) (Figure 8C).

### Activation of neurodegenerative and metabolic gene programs in SIV-infected hippocampus.

DGE analysis across control and SIV-infected ROIs (*n* =12 control; *n* = 12 SIV) demonstrated altered expression of numerous neurodegenerative and metabolic KEGG pathway genes in response to SIV; specifically, 52 metabolic and 25 neurodegenerative KEGG pathways were disrupted with SIV (Figure 8, D and E). We saw decreased expression of ETC genes (*NDUFB7*, *NDUFB11*, *COX4I1*) and a decrease in inositol polyphosphate phosphatase 4A (*INPP4A*), a suppressor of glutamate excitotoxicity in the CNS linked to neurodegeneration in the striatum. Additionally, we identified increase in *BST1*, a risk factor for neurodegenerative diseases (NDs). Downregulation of *HSP5*, *CTNNB1*, *COX4I1*, *KLC1*, *DCTN1*, and *PSMB7* linked to various neurodevelopmental and NDs was also observed (32). The upregulation of the mitochondrial calcium uniporter, located on the inner mitochondrial membrane, is noteworthy, as disturbances in calcium homeostasis are linked to ND (33–37).

### Single-cell analysis identifies activated inflammatory macrophage population in SIV-infected brain.

As spatial transcriptomics does not offer sc resolution, we complemented our analysis with sc gene expression of CD45^+^ enriched brain cells from control animals (33980, 33994), as described in Figure 2, in conjunction with SIV^+^ animal 32967. To delineate myeloid cell activation at a deeper resolution, we subclustered the myeloid cluster (macrophages and microglia) into 8 distinct subclusters. Utilizing established microglial markers (*PTPRC*, *ITGαM*, *CX3CR1*, *P2RY12*), cluster 3 and cluster 5 were designated to be like microglia (Figure 8F), while clusters 0, 1, 2, 4, and 7 expressed definitive macrophage markers *CD68* and *FCGR1A*. HLA genes *B2M* and *CD74* were primarily expressed in clusters 0, 2, 4, and 6. Genes related to antiviral responses (*IFIT2*, *IFIT3*, *IRF3*, *MAVS*, *STING1*, *TNF*) and chemokine trafficking (*CCL5*, *CCL19*, *CCL21*, *CCR5*, *CXCR3*) showed variability but were generally expressed at low levels. Cluster 4 was noteworthy, as it was enriched in the SIV brain. Cluster 4 displayed a gene signature of activated inflammatory macrophages, featuring high expression of MHC genes, and *IL-1β*. Assessment DGEs in total CD45^+^ cells showed alterations in chemokine, T cell receptor, MAPK signaling pathways due to chronic SIV infection. Key genes linked to T cell function (*STAT4*, *PTPN6*, *NFATC3*, *NFκB1*, *JAK3*, etc.) and MAPK signaling pathway (*EPHA2*, *PTPRR*, *TRAF2*, *NFATC3*) (*SPI1*, *NFATC3*, etc.) were altered (Figure 8G). In summary, spatial and sc analyses unveiled significant alterations in genes governing neuroinflammatory processes in both myeloid and T cells during chronic SIV infection.

### CCR7^+^ CD4^+^ T cell frequencies decreased during SIV-induced neuroinflammation.

**Figure 9:**
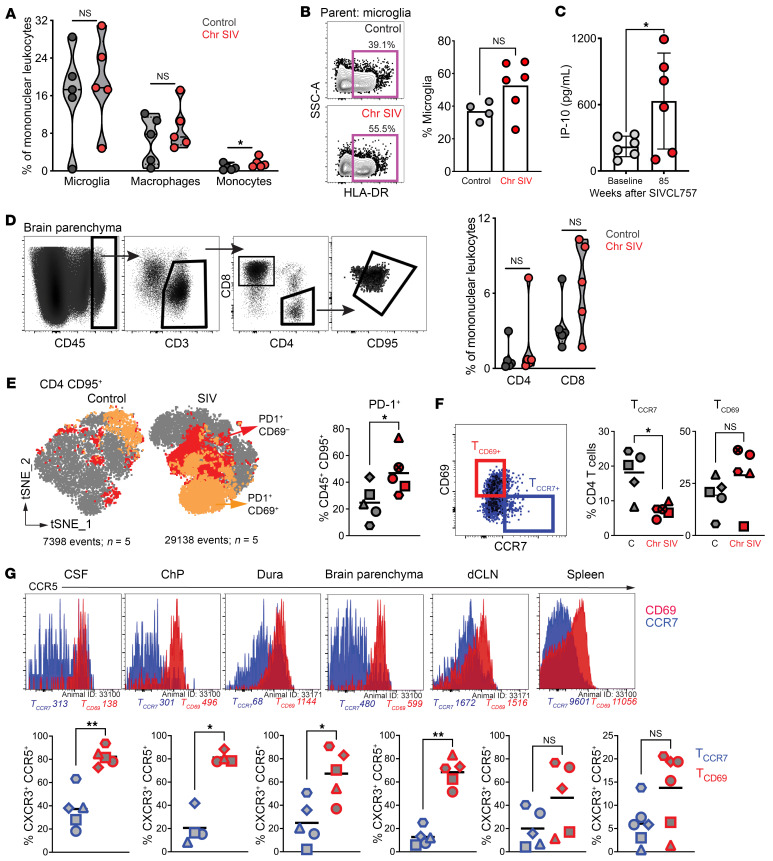
CCR7^+^ CD4^+^ T cell frequencies decreased during SIV-induced neuroinflammation. (**A**) Frequencies of myeloid (microglia, macrophage, and monocytes) within the control (black) and chronically infected SIV brain (red). (**B**) Representative flow plot illustrating HLA-DR expression on microglia cells (left); Frequency of HLA-DR expression (right). (**C**) IP-10 concentration within the rhesus CSF between baseline (grey) and chronic SIV CL757 infection (red; week 85). (**D**) Representative flow plots show CD4^+^ and CD8^+^ T cells in brain (left) and scatter plot shows frequencies (right). (**E**) t-SNE plot shows distribution of PD-1^+^ cells control and SIV brain (left) and scatter plot shows significantly higher PD-1^+^ frequencies with chronic SIV (right). (**F**) Representative flow plots depict gating strategy for T_CD69+_(red gate) and T_CCR7+_ (blue gate) populations (left); Frequencies of CCR7^+^ and CD69^+^ T cell populations (right) in the control (grey) and chronic SIV CL757 (red) infected brain. (**G**) Histogram plots indicating CCR5 expression and MFI (top) on T_CCR7_ (blue) and T_CD69_ (red); Frequencies of CXCR3^+^ CCR5^+^ within T_CCR7_ and T_CD69_ across the CSF, Choroid plexus (ChP), Dura, Brain Parenchyma, deep cervical lymph node (dCLN) and spleen. *P < 0.05, **P < 0.01.

After uncovering a complex interplay of genes involved in the initiation and progression of neuroinflammation in response to viral presence in the brain, we examined cellular and soluble markers in the CNS. We assessed myeloid populations (microglia [CD11b^+^ CD45^lo/int^], macrophages, monocytes [CD45^+^ CD14/CD16^+^]), and lymphoid populations (CD4^+^ and CD8^+^ T cells) from single cell suspensions. We observed a significant increase in brain monocytes, indicative of active recruitment to CNS during chronic SIV (Figure 9A). Correspondingly, there was a significant increase in plasma levels of CCL2, a monocyte chemoattractant (data not shown). We also investigated microglial activation in the brain and found that overall, HLA-DR expression on microglia was not significantly different (Figure 9B). To explore the potential for active recruitment of monocytes and/or lymphocytes to the brain, we examined a panel of inflammatory analytes. Interferon protein 10 (IP-10), a chemoattractant for monocytes and T cells, was significantly increased in the CSF at weeks 70–93 after infection, suggesting ongoing recruitment (Figure 9C). While the total CD4^+^ T cell population in the brain remained unchanged during chronic SIV (Figure 9D), a significant increase in the frequency of CD4^+^ T cells expressing PD-1 was noted, indicative of immune activation (Figure 9E).

Importantly, the CCR7^+^ CD69^–^ CD4^+^ subset was decreased in the brain during chronic SIV (Figure 9F), an effect not observed in adjacent CNS compartments (Supplemental Figure 13). To determine if the decrease in the CCR7^+^ CD4^+^ subset was a consequence of virus-mediated depletion, we examined expression of chemokine receptors CXCR3 and CCR5 expressed by target cells. The data showed that, on average, CD4^+^ CD69^+^ cells in the CNS expressed higher relative levels of CXCR3^+^ CCR5^+^ arguing against virus mediated depletion of CCR7^+^ CD4^+^ T cells (Figure 9G). In sum, the data emphasize the CNS immune surveillance role of CCR7^+^ CD4^+^ T cells and their potential to counter neuroinflammatory processes during chronic neuroinflammation.

## Discussion

Our data show that T cells in the noninflamed CNS exhibit diverse differentiation states, characterized by unique chromatin accessibility patterns corresponding to T_CM_ and T_EM_ and/or T_RM_ profiles. Beyond their presence in CSF and brain parenchyma, we also identify T_CM_ cells occupying the skull BM niche, potentially poised to replenish adjacent CNS compartments. Notably, impeding T cell egress from lymph nodes using FTY720 led to reduced CCR7^+^ CD4^+^ T cells within the CSF, suggesting potential migration of T_CM_ from lymph nodes to the CSF, likely through vascular routes. Lastly, in a chronic HIV infection model, we observed a specific decline in CCR7^+^ CD4^+^ T cells in the brain parenchyma.

While the presence of CCR7^+^ CD4^+^ T cells in the brain challenges established paradigms of memory T cell distribution in nonlymphoid tissues, there is precedence for our observations. For instance, 90% of CSF T cells express CCR7 (4, 38), and CCR7^+^ T cells populate nonlymphoid tissues including the skin, gut, colon, and cervix (39–41). Moreover, ligands for CCR7, namely CCL19 and CCL21, are present in human CNS (42–44). In rodent models, CCL19 and CCL21 production in the CNS is linked to CCR7^+^ CD8^+^ T cell recruitment (45). The reduction in CCR7^+^CD4^+^ T cells during chronic SIV infection could stem from multiple mechanisms: (a) CCR7 binding with CCL19 triggering its internalization, (b) Diminished CCR7 expression might result from the influx of SIV-specific CCR7^–^ effector cells or the effector differentiation of herpes-virus specific CD4^+^ memory cells in the brain parenchyma (38, 46–48) Notably, within the SAS, activated CCR7^–^ T cells enter the brain parenchyma, while quiescent CCR7^+^ T cells migrate out of the CNS (49). (c) CCR7^+^ cells might migrate to lymph nodes through nasal lymphatics (50), and (d) The HIV-1 viral protein U (Vpu) could downregulate CCR7, though this assumes widespread productive infection and likely does not fully account for the observed changes (51). Conclusively establishing the relative contributions of these factors holds significant implications for neuroinflammation.

Currently, it remains unclear whether increased neuroinflammation is a cause or a result of the loss of CCR7^+^ CD4^+^ T cells in the brain, or if these cells are direct targets of the virus. Thus, several key questions need to be addressed to establish the underlying mechanisms and pathological outcomes of this reduction. Firstly, the colocalization of virus with CD4^+^ T cells, as observed in the brain parenchyma with SIV mac251 (31, 52), still needs confirmation with SIVCL757. Secondly, increased PD-1^+^ CD4^+^ T cell frequencies in the chronic SIV-infected brain align with observed immune activation indices. However, there is a need for a more in-depth exploration of associated pathways of immune dysregulation, such as the imbalance of T_h_17 and Tregs and their potential pathogenic consequences (53).This is particularly relevant, as CCR7^+^ CD4^+^ T cells exhibit features of T_h_17 and T regulatory cells. Finally, our FTY720 studies suggest broad immune activation, raising questions about effects on CD4^+^ helper subsets like T regulatory cells (54) and connection to CNS immune activation.

In summary, we demonstrate that CD4^+^ T cells with T_CM_ features reside within the primate CNS. Taken together, these data support a model of CNS immunosurveillance by CCR7^+^ T_CM_-like population. During chronic viral infection, T_CM_-like cell frequencies are perturbed, likely due to egress to the draining deep cervical lymph node or differentiation to T_EM_ in response to local antigen. Further studies defining their migration potential and functional features will advance our understanding of neuroimmune surveillance during homeostasis and dysregulation in disease.

## Methods

### Sex as a biological variable.

Animals of both sexes were included in the study, except for the chronic SIV study, which consisted only of females. This decision was made because females are at a higher risk for neurodegenerative diseases, and we aimed to reduce variability in our infection studies.

### Rhesus macaques.

Colony-bred Rhesus Macaques (*Macaca mulatta*) were sourced and housed at the California National Primate Research Center (CNPRC). Animals (total *n* = 47) consisted of both males (*n* = 17) and females (*n* = 30) with ages ranging from 8 months to 29 years. Select animals were utilized for FTY720 treatment studies (*n* = 12) and SIV infection studies (*n* = 5). Additional tissues were obtained from uninfected opportunistic medical culls for unrelated conditions (*n* = 9; 4 males, 5 females; ages 8 months to 19 years 7 months) to bolster analyses with low sample sizes. Animal details are in Supplemental Tables 1 and 2.

### FTY720 treatment.

Fingolimod (FTY720) was obtained from Sigma-Aldrich and given orally daily by mixing with animal feed. Animals (*n* = 12, 10 males, 2 females; ages 3 years 5 months to 5 years 6 months) received 30 μg/kg of FTY720 daily over 4 weeks.

SIV infected animals were infected with SIVsm804e-CL757 strain (SIVCL757) donated by the Hirsch laboratory (NIAID, NIH, Bethesda, Maryland, USA) (30). Viral stocks were thawed at 4°C and diluted 16.7-fold in HBSS to a final volume of 0.5 mL and stored on ice before administration. Animals (*n* = 6; 6 females; ages 17 years 6 months to twenty years 8 months were intravenously infected with 500 TCID_50_. Animals were treated with ART regimen consisting of Emtricitabine [40 mg/kg], Tenofovir Disproxil Fumarate [5.1 mg/kg,] and Dolutegravir [2.5 mg/kg]) administered as previously described (31).

### Specimen collection and processing.

Animals were anesthetized with 10 mg/kg of ketamine hydrochloride administered intramuscularly for routine collections and with pentobarbital at necropsy. Collection of plasma, serum, and PBMCs were performed as previously described (55). CSF was collected via the foramen magnum and examined for blood contamination by both visual inspection and Hemastix testing strips (Siemens) in accordance with manufacturer instructions. Lymphoid tissues and CNS-associated tissues were obtained at necropsy following cardiac saline perfusion and immediately processed for isolation of mononuclear cells using collagenase IV digestion and a 21%/75% percoll gradient.

### Flow cytometry.

Whole blood, CSF, and fine-needle lymph node aspirates (FNA) were freshly stained and acquired on the same day following collection. Mononuclear cells obtained from necropsy tissues were either freshly stained and acquired the same day or stained following cryopreservation, with identical methods used for all comparisons. Antibody information can be found in Supplemental Table 3. Sample acquisition and fluorescence measurements were performed on a BD Bioscience FACSymphony utilizing FACSDiva software (Version: 8.0.1). Sample compensation, population gating, and analysis was performed using FlowJo (Version 10.8.1).

### Intracellular Cytokine Stimulation Assay.

Aliquots of 2 million freshly collected PBMCs and CSF cells were stimulated with PMA/Ionomycin (eBioscience Cell Stimulation Cocktail) and incubated for 1 hour at 37°C. Brefeldin A (BD GolgiPlug) and monensin (BD GolgiStop) was added to cell suspensions and incubated at 37°C for an additional 4 hours. The remainder of the procedure was carried out as previously described (56).

### FlowSOM.

Clustering of cells and construction of a minimum spanning tree of relationships between clusters was conducted using FlowSOM, version 2.4.0 (57) with logicle transformation (58). Clustering was based on CXCR3, CD95, PD-1, CD69, and CCR5, and with CCR7 expression used to define groups within metaclusters. Numbers of metaclusters were selected dynamically by the FlowSOM algorithm. Data from each panel were analyzed separately with a cluster defined as overrepresented in the CCR7^+^ or CCR7^–^ group, if the corresponding adjusted standardized residual from the χ^2^ test performed on the table of cluster cell counts by CCR7 status was greater than 3. Analyses were conducted using R version 4.2.1.

### vRNA quantification.

Quantification of plasma and CSF vRNA was performed as previously described, using a quantitative reverse-transcriptase PCR (qRT-PCR) assay for the detection of SIV gag (59).

### Cell preparation for sequencing studies.

Cryopreserved mononuclear cells from rhesus brain and splenic tissues were thawed at room temperature, placed in fresh complete media (For splenic cells, RPMI supplemented with 10% HI-FBS, 1% L-glutamine, 1% penicillin-streptomycin; For brain tissue derived cells, DMEM supplemented with 10% HI-FBS, 1% L-glutamine, 1% penicillin-streptomycin [Sigma-Aldrich]) and treated with 2 units/mL of DNase I (Roche Diagnostics) for 15 minutes at 37°C. Cells were washed in complete medium and CD45^+^ cells were isolated using CD45 microbeads for nonhuman primates (Miltenyi Biotech) in accordance with the manufacturer’s protocol. Enriched CD45^+^ cells were stained for CD45 and a live-dead marker for subsequent flow cytometric sorting. Live CD45^+^ cells were characterized and quantified on a BD FACSymphony cell analyzer, sorted utilizing a FACS Ari,a and suspended in DMEM for scRNA-Seq studies.

### scRNA-Seq.

Sample processing, including barcoding, gel-bead assembly in emulsion (GEM), GEM reverse transcription, cDNA amplification, and library construction, followed the Chromium Next GEM single cell 3′ v3.1 protocol by 10X Genomics. Sequencing and bioinformatic analysis was performed as previously described (31). DGE analysis across different cell types and conditions was conducted using Seurat functions, employing a threshold of (*P*_adj_ < 0.05, |log_2_ FC| > 0.25) with Benjamini-Hochberg correction. Gene-set enrichment analysis and functional annotation were carried out using clusterProfiler 4.0 and visualized through custom scripts. Pathways represented by DGE genes were visualized with a chord plot utilizing the ‘circlize’ package in R. All subsequent data analysis was performed in R v4.2.0.

### snRNA-Seq and ATAC.

Nuclei were isolated from brain-derived live CD45^+^ and CD45^–^ cells using the Chromium Nuclei Isolation Kit (10X Genomics) using the manufacture’s instructions. Following isolation, nuclei were stored on ice and used immediately for subsequent library preparation. A barcoded 3′ sc gene expression library and ATAC library were prepared from single-nuclei suspensions using the Chromium Single Cell Multiome kit (10X Genomics) following the manufacturer’s instruction. The libraries were quantified by fluorometry on a Qubit instrument (LifeTechnologies) and by qPCR with a Kapa Library Quant kit (Kapa Biosystems-Roche) prior to sequencing. The libraries were sequenced on a NovaSeq 6000 sequencer (Illumina) with paired-end 150 bp reads with approximately 35,000 reads pairs per nuclei for gene expression and 25,000 read pairs per nuclei for ATAC libraries.

### snRNA-Seq and ATAC quantification and statistical analysis.

The raw sc multiome (ATAC + Gene Expression) sequencing data were preprocessed using Cell Ranger ARC pipeline (10X Genomics). This step involved the demultiplex of cells using cell barcodes, the alignment of reads to Mmul10 reference genome, removal of empty droplets, cell debris, and low-quality cells. The filtered gene expression data was imported to Seurat (60) (v4.3.0) for further quality control. Cells were required to have between 250 and 5,000 genes, 500 and 12,000 unique transcripts, and 1,000 and 70,000 ATAC peaks. Cell doublets were removed using DoubletFinder (61). Gene expression data and chromatin accessibility data were normalized, and dimensionality was reduced individually using LogNormalize method in Seurat with a scale factor of 10,000. Chromatin accessible peak data produced by Cell Ranger was processed using Signac (62) (v1.8.0) and normalized using term frequency-inverse document frequency (TF-IDF) in RunTFIDF function, selecting the top features and then dimension reduced using singular value decomposition on the TF-IDF matrix. After dimension reduction, the 2 modalities were integrated using the weighted nearest neighbor method in Seurat. The integrated graph was then used for UMAP visualization and clustering. Cell type identification was carried out on clusters generated at resolution 2.25, using the R package scType (63). Differential expression analysis was done using a linear model in limma (64) and adjusting for cell cycle scores and the number of genes expressed.

### Spatial transcriptomics profiling.

Paraformaldehyde-fixed, paraffin-embedded hippocampal brain tissues were profiled using GeoMx DSP (65). Tissue sections of 5 μm were prepared according to manufacturer’s recommendations for GeoMx-NGS RNA BOND RX slide preparation (manual no. MAN-10131-02). Tissue morphology was visualized using fluorescent antibodies specific to lymphocyte and neuron-specific markers (anti-CD45 [Novus], anti-CD3 [Primary, Bio-Rad], Secondary anti-Rat IgG, [Thermo Fisher Scientific], and anti-NeuN [Sigma-Aldrich], and nucleic acid stain Cyto83 [Thermo Fisher Scientific]). Twelve ROIs of 660 × 785 μm geometric shapes (squares) were created and localized to brain blood vessels and neuron-rich areas. After ROI selection, UV light was utilized to release and collect oligonucleotides from each ROI. For Whole Transcriptome analysis, Illumina i5 and i7 dual-indexing primers were added to each area of illumination (AOI) during PCR. Library concentration was measured using a Qubit fluorometer (Thermo Fisher Scientific), and quality assessed using a Bioanalyzer (Agilent). The Illumina NextSeq 2000 was used for sequencing, and the resulting FASTQ files were mapped to the Hs_R_NGS_WTA_v1.0 reference (Nanostring) using the NanoString GeoMx NGS pipeline v2.1 to generate raw count data for each target probe AOIs. Raw counts were processed using the same NanoString GeoMx NGS pipeline and converted to digital count conversion (DCC) files. For protein analysis, oligos were enumerated on the nCounter platform. Protein data was normalized to ERCC-Seq specific probes followed by area normalization, and lastly to control IgG (rabbit and mouse) to control for background signal.

DCC files were further processed using Geomxtools (66) R package (Bioconductor version 3.2.0, Nanostring). Data were quality controlled per individual AOI. AOIs were excluded from the data set if they met any of the following conditions: less than 80% reads aligned to the reference, less than 40% sequencing saturation, or less than 1,000 raw reads. Limit of quantification (LoQ) calculated for raw data based on the distribution of the negative control probes (“NegProbe”) and used as an estimate for the quantifiable limit of gene expression per AOI (67). A gene was considered detected if its expression was above the LoQ for that AOI. Genes were included in the analysis if they were detected above the LoQ in over 5% of AOIs. Then, the data was normalized using the third quantile (Q3) to account for differences in cellularity and ROI size. The Linear Mixed Model (LMM) was used to calculate the significant differences between the 2 groups, and genes were considered significantly expressed when *P*_adj_ < 0.1 (677 DGEs). The differentially expressed genes were used to create heatmaps of selected KEGG pathways. The heatmaps were created using ComplexHeatmap (version 2.13.1) R package (68).

### IHC.

Deidentified formalin-fixed paraffin embedded human hippocampal tissues from patients who were nondemented (*n* = 3) and a patient with glioblastoma (*n* = 1) were obtained from the Netherlands Brain Bank, and human tonsil tissues (*n* = 1) were obtained from the UC Davis Cancer Center Repository. Rabbit polyclonal anti-CD3 (Agilent; A045229-2), mouse monoclonal anti-CD4 (Novus; NBP2-46149), rabbit polyclonal anti-CD45 (Abcam, AB10558), rabbit monoclonal anti-IBA1 (Invitrogen, MA5-36257), rabbit polyclonal anti-CD11b (Invitrogen, PA5-79533), and rabbit monoclonal anti-NeuN (Abcam, AB177487) were used for subsequent IHC staining. All 4 μm paraffin sections were subjected to a heat antigen retrieval step before application of primary antibodies by treating slides with AR-10 (Biogenex) for 2 minutes at 125°C in a Digital Decloaking Chamber (Biocare), followed by cooling to 90°C for 10 minutes, or by treating slides with H-3300 (Vector) for 20 minutes at 100°C. Following primary staining, samples were incubated with anti-mouse and anti-rabbit EnVision+ system secondary antibodies (Agilent, K400111-2 and K400311-2), followed by treatment with AEC chromogen (Agilent) and counterstained with Gill’s hematoxylin I (StatLab). Primary antibodies were replaced by mouse or rabbit isotype controls and run with each staining series as negative controls. Slides were visualized with a Zeiss Imager Z1 (Carl Zeiss) and images captured using a Zeiss Axiocam (Carl Zeiss).

### Statistics.

Wilcoxon signed rank tests were used for paired analyses (i.e., longitudinal and within group comparisons). Mann-Whitney *U* tests were used for unpaired comparison between animal cohorts/treatment groups. Tests were performed in GraphPad Prism (Version 9.5.1) with significance values denoted as follows: *P < 0.05, **P < 0.01, ***P < 0.001, ****P < 0.0001.

### Study approval.

All nonhuman primates were maintained in accordance with American Association for Accreditation for Laboratory Animal Care (AAALAC) and Animal Welfare Act guidelines and according to the guidelines of the IACUC of University of California, Davis. All procedures were approved by the Institutional Animal Care and Use Committee (IACUC) at UC Davis (protocol nos. 22379, 22261, 22787, 22033, 23363).

### Data availability.

The scRNA-Seq data set is accessible at GSE221815. snRNA + ATAC data set accessible at GSE261968. Spatial transcriptomics RNA-seq data set is accessible at GSE261969. All data are provided in the Supporting Data Values file.

## Author contributions

SRE, AV, YSL, SSI, and JHM designed the studies. SSI, J Lifson, and RR supervised experiments. SRE, AV, YSL, ZMM, SSI, DB, PBP, and SO performed experiments. SRE, YSL, J Li, BPDJ, ARD, BTS, DR, and SSI analyzed data. SSI, SRE, and CEH wrote and revised the manuscript. All authors read, edited, and approved the final manuscript.

## Supplementary Material

Supplemental data

Supporting data values

## Version 1 03/12/2024

In-Press Preview

## Version 2 05/01/2024

Electronic publication
