# Supplementary material for: Chronic SIV-Induced neuroinflammation disrupts CCR7+ CD4+ T cell immunosurveillance in the rhesus macaque brain
Source: J Clin Invest. 2024 Mar 12;134(9):e175332. doi: 10.1172/JCI175332 (PMC11060742; doi:10.1172/JCI175332)
Supplement: Supplemental data [file jci-134-175332-s130.pdf]

S1 Table. NHP cohort for chronic SIV study (SIVCL757)

Animal ID	Infection status	Age at Euthanasia(years:months:day)	Sex	Weight (kg)	TRIM5	ART regimen
33100	Uninfected	20:07:09	**F**	**12.32**	TFP/Q	N/A
33171	Uninfected	20:06:27	F	9.27	TFP/TFP	N/A
33980	Uninfected	19:06:26	F	9.6	TFP/TFP	N/A
33994	Uninfected	19:06:16	F	10.31	TFP/TFP	N/A
34194	Uninfected	19:06:16	F	11.84	TFP/TFP	N/A
35886	SIVCL757	17:05:27	F	9.11	TFP/Q	FTC/TDF/DTG
35595	SIVCL757	17:07:17	F	11.9	Q/CypA	FTC/TDF/DTG
34974	SIVCL757	17:08:22	F	8.94	TFP/Q	FTC/TDF/DTG
32967	SIVCL757	20:07:21	F	13.06	TFP/TFP	FTC/TDF/DTG
33191	SIVCL757	20:07:02	F	11.06	TFP/TFP	FTC/TDF/DTG
34996	SIVCL757	18:06:20	F	11.46	N/A	FTC/TDF/DTG

SIV: Simian Immunodeficiency Virus; ART: Anti-Retroviral Therapy; FTC: Emtricitabine; TDF: Tenofovir disoproxil fumarate; DTG: Dolutegravir.

S2 Table. Nonhuman primate cohort for tissue assessment during medculls/necropsies

Animal ID	Sex	Age (years.months)	Weight (kg)	Infection status	Study Treatments	Euthanasia timepoint at weeks post-infection	Medcull Condition
38163	F	15.07	7.48	Uninfected	N/A	N/A	N/A
40691	M	12.08	12.18	Uninfected	N/A	N/A	N/A
38691	F	14.11	13.56	Uninfected	N/A	N/A	N/A
40499	F	12.11	9.79	Uninfected	N/A	N/A	N/A
45721	M	5.06	14.75	Uninfected	FTY720	N/A	N/A
45781	M	5.06	8.66	Uninfected	FTY720	N/A	N/A
46235	M	4.07	14	Uninfected	FTY720	N/A	N/A
46354	M	4.06	10.96	Uninfected	FTY720	N/A	N/A
46410	M	4.06	12.7	Uninfected	FTY720	N/A	N/A
46548	M	4.05	8.25	Uninfected	FTY720	N/A	N/A
46551	M	4.05	8.59	Uninfected	FTY720	N/A	N/A
47081	F	3.06	5.28	Uninfected	FTY720	N/A	N/A
47154	M	3.06	8.63	Uninfected	FTY720	N/A	N/A
47161	M	3.06	9.32	Uninfected	FTY720	N/A	N/A
47387	F	3.05	7.85	Uninfected	FTY720	N/A	N/A
47466	M	3.04	7.04	Uninfected	FTY720	N/A	N/A
34051	F	20.00	6.97	Uninfected	N/A	N/A	N/A
35488	F	18.01	8.54	Uninfected	N/A	N/A	N/A
36798	F	16.11	10.31	Uninfected	N/A	N/A	N/A
37164	F	16.01	10.83	Uninfected	N/A	N/A	N/A
40234	F	12.11	8.72	Uninfected	N/A	N/A	N/A
40742	M	12.00	11.81	Uninfected	N/A	N/A	N/A
41217	F	11.11	10.77	Uninfected	N/A	N/A	N/A
41812	F	11.00	6.51	Uninfected	N/A	N/A	N/A
25888	F	29.01	9.92	Uninfected	N/A	N/A	N/A
33374	F	19.08	6.64	Uninfected	N/A	N/A	N/A
35328	M	17.03	11.22	Uninfected	N/A	N/A	N/A
47862	M	0.08	1.27	Uninfected	N/A	N/A	CHRONIC DIARRHEA
42318	F	10.11	8.25	Uninfected	N/A	N/A	NX (UNRELATED PROJECT)
39157	F	11.08	8.88	Uninfected	N/A	N/A	BILATERAL RENAL FAILURE
35115	M	16.08	12.12	Uninfected	N/A	N/A	BILATERAL ARTHRITIS STIFLES
44003	F	5.11	5.5	Uninfected	N/A	N/A	BILATERAL ARTHRITIS STIFLES
44510	M	5.09	7.47	Uninfected	N/A	N/A	REGENERATIVE JOINT DISEASE - STIFLES
44288	F	5.01	4.9	Uninfected	N/A	N/A	TRICHOBEZOAR; CHRONIC ULCERATIVE GASTRITIS
41647	M	8.11	9.18	Uninfected	N/A	N/A	HEPATIC AMYLOID
46078	F	19.07	6.19	Uninfected	N/A	N/A	HEMOABDOMEN
33100	F	20.07	12.32	Uninfected	N/A	N/A	N/A
33171	F	20.07	9.27	Uninfected	N/A	N/A	N/A
33980	F	19.07	9.6	Uninfected	N/A	N/A	N/A
33994	F	19.06	10.31	Uninfected	N/A	N/A	N/A
34194	F	19.06	11.84	Uninfected	N/A	N/A	N/A
35886	F	17.06	9.11	SIVCL757	Embicitrabine, Tenofovir Disproxil Fumarate, Dolutegravir (ART)	121	N/A
35595	F	17.08	11.9	SIVCL757	Embicitrabine, Tenofovir Disproxil Fumarate, Dolutegravir (ART)	121	N/A
34974	F	17.09	8.94	SIVCL757	Embicitrabine, Tenofovir Disproxil Fumarate, Dolutegravir (ART)	78	BICAVITARY EFFUSION
32967	F	20.08	13.06	SIVCL757	Embicitrabine, Tenofovir Disproxil Fumarate, Dolutegravir (ART)	121	N/A
33191	F	20.07	11.06	SIVCL757	Embicitrabine, Tenofovir Disproxil Fumarate, Dolutegravir (ART)	121	N/A
34996	F	18.07	11.46	SIVCL757	Embicitrabine, Tenofovir Disproxil Fumarate, Dolutegravir (ART)	121	N/A

S3 Table. Key reagents and resources

REAGENT or RESOURCE	SOURCE	IDENTIFIER
Antibodies		
Mouse anti-Human CXCR3 (Clone:1C6/CXCR3) - APC	BD Biosciences	Cat# 550967; RRID: AB_398481
Mouse anti-Human CD16 (Clone:3G8) - APC	BD Biosciences	Cat# 561248; RRID: AB_10612010
Mouse anti-Human CD49d (Clone: HP2/1) - APC	Beckman Coulter	Cat# B01682; RRID: AB_398681
Mouse anti-Human CD28 (Clone: 28.2) - APC-Cy7	BioLegend	Cat# 302966; RRID: AB_2800753
Mouse anti-Human CD3 (Clone: SP34-2) - APC-Cy7	BD Biosciences	Cat# 557757; RRID: AB_396863
Mouse anti-Human CD20 (Clone: 2H7) - APC-Cy7	BioLegend	Cat# 302314; RRID: AB_314262
Mouse anti-Human KI-67 (Clone: B56) - Alexa Fluor 488	BD Biosciences	Cat#558616; RRID: AB_10611866
Mouse anti- Non-Human Primate CD45 (Clone: D058-1283) - Alexa Fluor 488	BD Biosciences	Cat# 557803; RRID: AB_396879
Mouse anti-Human BCL2 (Clone: Bcl-2/100) - Alexa Fluor 647	BD Biosciences	Cat# 563600; RRID: AB_2738306
Mouse anti-Human IL-21 (Clone:3A3-N2.1) - Alexa Fluor 647	BD Biosciences	Cat# 560493; RRID: AB_1645421
Mouse anti-Human CD3 (Clone: SP34-2) - Alexa Fluor 700	BD Biosciences	Cat#557917; RRID: AB_396938
Mouse anti-Human CD14 (Clone: M5E2) - Alexa Fluor 700	BioLegend	Cat# 301822; RRID: AB_493747
Rat anti-Human CCR7 (Clone:3D12) - PE	BD Biosciences	Cat# 552176; RRID: AB_394354
Mouse anti-Human CD163 (Clone: GHI/61) - PE	BioLegend	Cat# 333606; RRID: AB_1134002
Mouse anti-Human IL-21 (Clone:3A3-N2.1) - PE	BD Biosciences	Cat# 562042; RRID: AB_10896123
Mouse anti-Human CD21 (Clone: Bly-4) - PE-Cy7	BD Biosciences	Cat# 561374; RRID: AB_10681717
Mouse anti-Human CD103 (Clone: Ber-Act8) - PE-Cy7	BioLegend	Cat# 350212; RRID: AB_10782579
Mouse anti-Human CD11C (Clone: 3.9) - PE-Cy7	Invitrogen	Cat# 25-0116-42; RRID: AB_1582274
Mouse anti-Human EOMES (Clone: WD1928) - PE-Cy7	ThermoFisher	Cat# 25-4877-42; RRID: AB_2573456
Mouse anti-Human CD127 (Clone: eBioRDR5) - PE-Cy7	ThermoFisher	Cat# 25-1278-42; RRID: AB_1659672
Mouse anti-Human IFN-g (Clone: B27) - PE-Cy7	BioLegend	Cat# 506518; RRID: AB_2123321
Mouse anti-Human CCR6 (Clone: G034E3) - PE-CF594	BioLegend	Cat# 353430; RRID: AB_2564233
Mouse anti-Human CD40 (Clone: 5C3) - PE-CF594	BioLegend	Cat# 334342; RRID: AB_2566457
Mouse anti-Human IL-2 (Clone:MQ1-17H12) - PE-CF594	BioLegend	Cat# 500344; RRID: AB_2564091
Mouse anti-Human TNFA (Clone: MAb11) - FITC	BioLegend	Cat# 502906; RRID: AB_315258
Mouse anti-Human CXCR3 (Clone: 1C6/CXCR3) - BV421	BD Biosciences	Cat# 562558; RRID: AB_2737653
Mouse anti-Human FOXP3 (Clone: 206D) - BV421	Biolegend	Cat# 320124; RRID: AB_2565972
LIVE/DEAD Fixable Aqua Dead Cell Stain Kit - BV510	Life Technologies	Cat# L34966; RRID: N/A
Mouse anti-Human CD11b (Clone: ICRF44) - BV510	BD Biosciences	Cat# 563088; RRID: AB_2737996
Mouse anti-Human CD8 (Clone: SK1) - BV510	BD Biosciences	Cat# 563919; RRID: AB_2722546
Mouse anti-Non-Human Primate CD45 (Clone: D058-1283) - BV605	BD Biosciences	Cat# 564098; RRID: AB_2738590
Mouse anti-Human CCR4 (Clone: 1G1) - BV605	BD Biosciences	Cat# 562906; RRID: AB_2737882
Mouse anti-Human CD4 (Clone: L200) - BV650	BD Biosciences	Cat# 563737; RRID: AB_2687486
Mouse anti-Human CCR5 (Clone: 3A9) - BV650	BD Biosciences	Cat# 564999; RRID: AB_2739037
Mouse anti-Human CD69 (Clone: FN50) - BV711	BioLegend	Cat# 310944; RRID: AB_2566466
Rat anti-Human CX3CR1 (Clone:2A9-1) - BV711	BioLegend	Cat# 341630; RRID: AB_2814256
Mouse anti-Human CD95 (Clone: DX2) - BUV737	BD Biosciences	Cat# 564710; RRID: AB_2738907

Rat anti-Human CX3CR1 (Clone:2A9-1) - BV711	BioLegend	Cat# 341630; RRID: AB_2814256
Mouse anti-Human CD95 (Clone: DX2) - BUV737	BD Biosciences	Cat# 564710; RRID: AB_2738907
Armenian Hamster anti-Human/Mouse/Rat ICOS (Clone: C398.4A) - BV785	BioLegend	Cat# 313534; RRID: AB_2629729
Mouse anti-Human CCR5 (Clone: 3AG) - BV786	BD Biosciences	Cat# 565001; RRID: AB_2739039
Mouse anti-Human HLA-DR (Clone: L243) - BV786	BioLegend	Cat# 307642; RRID: AB_2563461
Mouse anti-Human CD8 (Clone: SK1) - BUV805	BD Biosciences	Cat# 564913; RRID: AB_2833078
Mouse anti-Human PD-1 (Clone: EH12.2H7) - Pacific Blue	BioLegend	Cat# 329916; RRID: AB_2283437
LIVE/DEAD Fixable Near-IR Dead Cell Stain Kit	Life Technologies	Cat# L34976; RRID: N/A
Mouse anti-Human CD45 (Clone: 2B11+PD7/26)	Novus	Cat# NBP2-34528AF647; RRID: AB_960384
Rat anti-Human CD3 (Clone: CD3-12)	Bio-Rad	Cat# MCA1477; RRID: AB_321245
Rabbit anti-NeuN (Clone: polyclonal)	Millipore Sigma	Cat# ABN78; RRID: AB_10807945
Goat anti-Rat IgG (H+L) Crossed Adsorbed secondary Antibody, Alexa Fluor 594	ThermoFisher	Cat# A-11007; RRID: AB_10561522
Syto83	ThermoFisher	Cat# S11364; RRID: N/A
Rabbit anti-Human CD3 (Clone: polyclonal)	Agilent	Cat# A045229-2; RRID: AB_2335677
Mouse anti-Human CD4 (Clone: OTI5D9)	Novus	Cat# NBP2-46149; RRID: N/A
Rabbit anti-Human CD11b (polyclonal)	Invitrogen	Cat# PA5-29633; RRID: AB_2547108
Rabbit anti-Human CD45 (Clone: polyclonal)	Abcam	Cat# ab10558; RRID: AB_442810
Rabbit anti-Human IBA1 (Clone: HL22)	Invitrogen	Cat# MA5-36257; RRID: AB_2890455
Rabbit anti-Human NeuN (Clone: EPR12763)	Abcam	Cat# ab177487; RRID: AB_2532109
Purified NA/LE Mouse anti-Human CD49d (Clone: 9F10)	BD Biosciences	Cat# 555501; RRID: AB_2130052
Purified NA/LE Mouse anti-Human CD28 (Clone: CD28.2)	BD Biosciences	Cat# 555725; RRID: AB_396068
**Bacterial and virus strains**		
SIVsm804e-CL757	Dr. Vanessa Hirsch, NIAID	doi: 10.1371/journal.ppat.1006538
**Biological samples**		
Rhesus macaque biological fluids (Blood and Cerebrospinal Fluid)	CNPRC, UC Davis	N/A
Rhesus macaque tissues (Brain tissue, pituitary gland, dura mater, choroid plexus, skull bone marrow, draining cervical lymph node, thoracic lymph node, fine needle axillary lymph node aspirates, spleen)	CNPRC, UC Davis	N/A
Formalin fixed and paraffin embedded human hippocampal tissue from non-demented anonymous human patients	Netherlands Brain Bank	N/A
Formalin fixed and paraffin embedded human hippocampal tissue from an anonymous human patient with glioblastoma	Netherlands Brain Bank	
Formalin Fixed and paraffin embedded human tonsil tissue	Cancer Center Repository, UC Davis	N/A

**Chemicals, peptides, and recombinant proteins**		
Brilliant Stain Buffer plus	BD Biosciences	Cat# 566385
BD FACS Lysing Solution	BD Biosciences	Cat# 349202
BD Cytofix/CytoPerm	BD Biosciences	Cat# 51-2090KZ
BD Perm/Wash	BD Biosciences	Cat# 51-2091KZ
eBioscience Foxp3/Transcription staining buffer set	Invitrogen	Cat# 00-5523-00
Protein Transport Inhibitor Containing Brefeldin A (BD GolgiPlug)	BD Biosciences	Cat# 555029
Protein Transport Inhibitor Containing Monensin (BD GolgiStop)	BD Biosciences	Cat# 554724
Gill's Hemotoxylin I	StatLab	Cat# HXGH1LT
Collagenase Type 4 (265 u/mg dw)	Worthington Biochemical Corporation	Cat# LS004188
DNase I Recombinant, RNase-free	Roche Diagnostics	Cat# 04716728001
Embicitrabine	Gilead Sciences	Cat# GS-9019
Tenofovir Disproxil Fumarate	Gilead Sciences	Cat# GS-4331
Dolutegravir (GSK Comet)	GSK plc	Cat# GSK1349572A
Hydroxypropylbetadex (Kleptose HPB)	Roquette	Cat# 346113102B
FTY720	Millipore Sigma	Cat# SMLO700
**Critical commercial assays**		
T cell activation/Expansion Kit, non-human primate	Miltenyi Biotec	Cat# 130-092-919
eBioscience Cell Stimulation Cocktail	Invitrogen	Cat# 00-4970-93
LEGENDplex NHP Inflammation Panel (13-plex)	BioLegend	Cat# 740389
CD45 microbeads, non-human primate	Miltenyi Biotec	Cat# 130-091-899
Chromium Next GEM Single Cell 3' GEM, Library & Gel Bead Kit v3.1	10X Genomics	Cat# PN-1000121
Chromium Nuclei Isolation Kit with RNase Inhibitor	10X Genomics	Cat# PN-1000494
Chromium Next GEM Single Cell Multiome ATAC + Gene Expression Reagent Bundle	10X Genomics	Cat# PN-1000283
**Deposited data**		
Single Cell RNA Sequencing Results	This paper	GEO Accession: GSE221815
**Experimental models: Organisms/strains**		
Rhesus macaque (Macaca Mulatta)	CNPRC, UC Davis	N/A
**Oligonucleotides**		
**Software and algorithms**		
GraphPad Prism (Version 9.5.1)	GraphPad	https://www.graphpad.com/
FACS Diva (Version 8.0.1)	BD Biosciences	https://www.bdbiosciences.com/en-us/products/software/instrument-software
FlowJo (Version 10.8.1)	FlowJo LLC	https://www.flowjo.com/
FlowSOM (Version 2.4.0)	Van Gassen et al. (doi: 10.1002/cyto.a.22625.)	https://bioconductor.org/packages/release/bioc/html/FlowSOM.html
R (Version 4.2.1)	R Core Team	https://www.r-project.org/
CellRanger (Version 7.0.1)	10X Genomics	https://support.10xgenomics.com/single-cell-gene-expression/software/pipeli
Seurat (Version 4.3.0)	Hao et al. (doi: 10.1016/j.cell.2021.04.048.); Stuart et al. (doi: 10.1016/j.cell.2019.05.031)	https://satijalab.org/seurat/

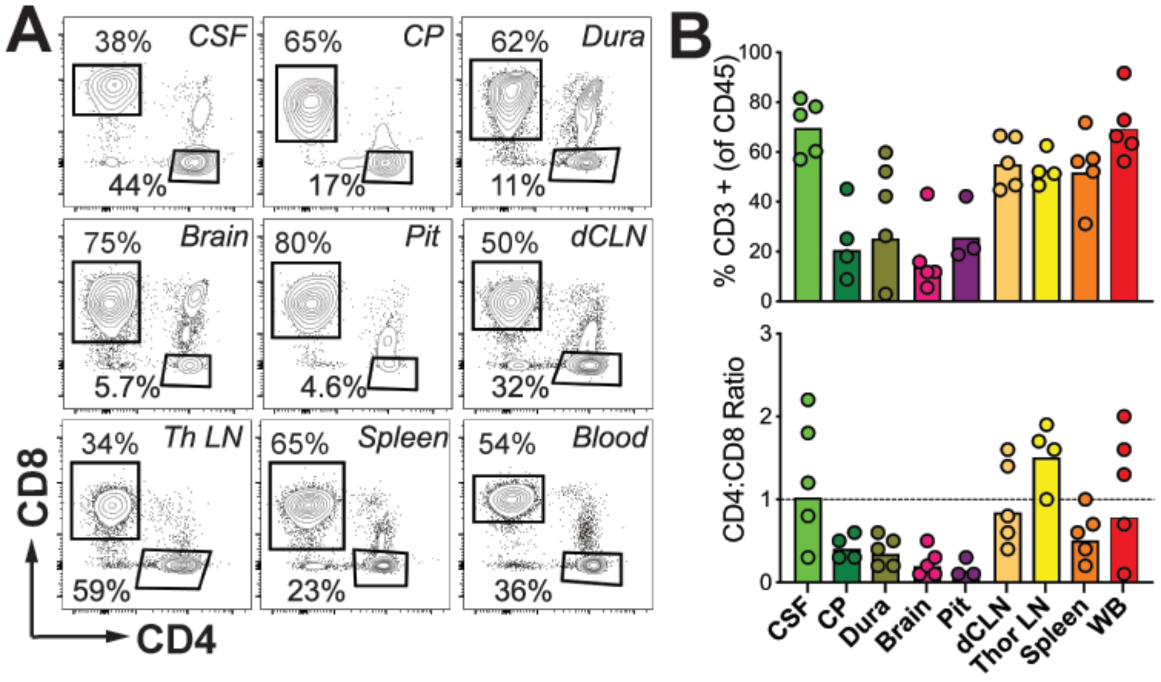

Figure S1. Distribution of CD4 and CD8 T cells in brain parenchyma and its border tissues, **Related to Figure 1** (**A**) Illustrates CD4 and CD8 T cell frequencies (gated on CD3+ cells) in various tissues as indicated. CSF, cerebrospinal fluid; CP, choroid plexus; Dura, Dura mater; Pit, Pituitary; dCLN, deep cervical lymph nodes, Th LN, thoracic lymph nodes. (**B**) bar graphs show % CD3+ T lymphocytes and CD4: CD8 ratios across CNS and lymphoid tissues.

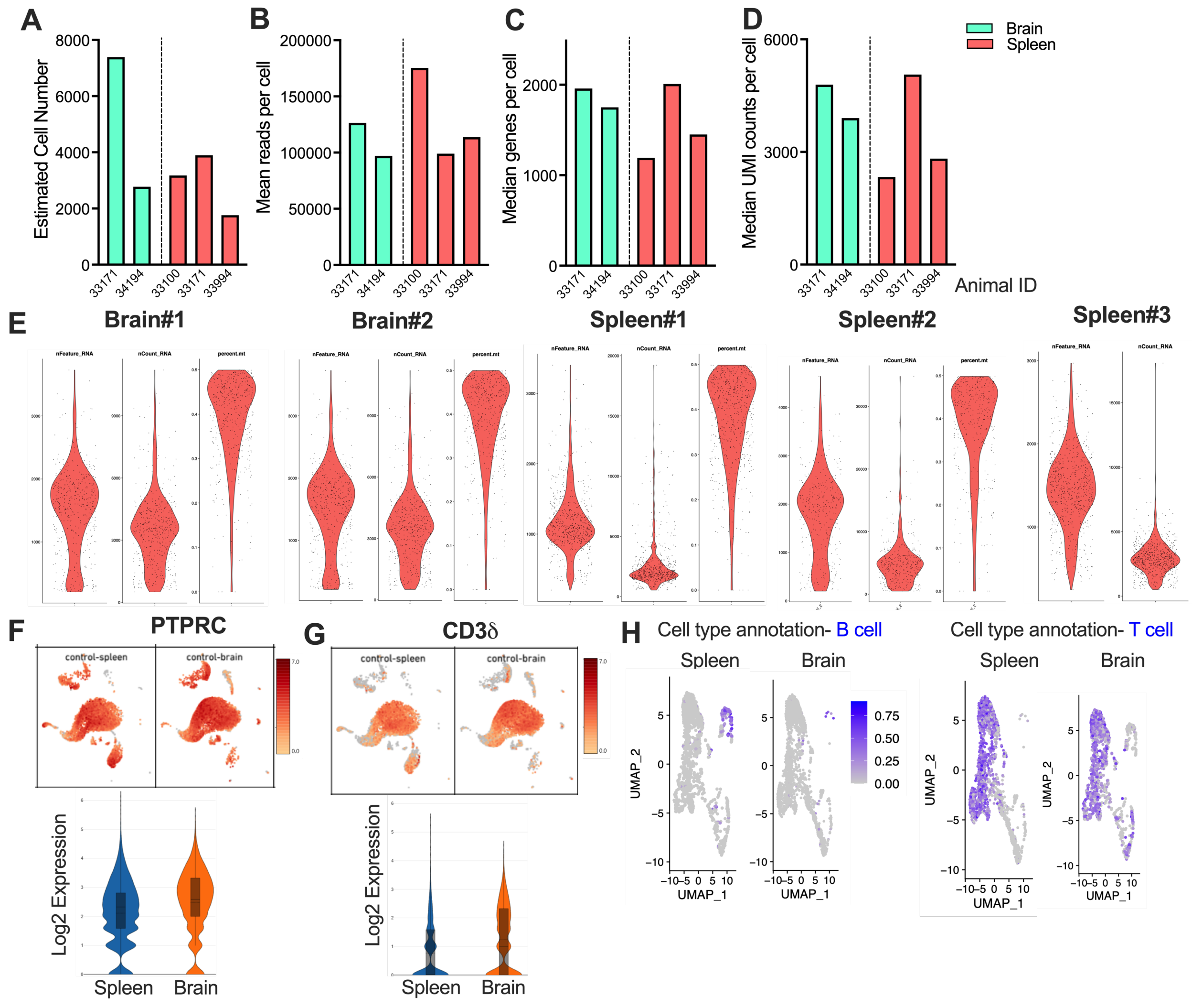

Figure S2. Single-cell transcriptomic analyses of CD45+ immune cells reveal presence of core T cell molecular programs in brain, related to Figure 1. (**A**) lists cell numbers sequenced for each sample. (**B-D**) Mean, median reads per cell and UMI counts per cell. (**E**) quality metrics on each sample. (**F**) Expression profile of PTPRC shown in UMAPs and violin plots. (**G**) Expression profile of CD3δ subunit shown in UMAPs and violin plots. (**H**) UMAP plots show cell type annotation for B cells and T cells across tissue types.

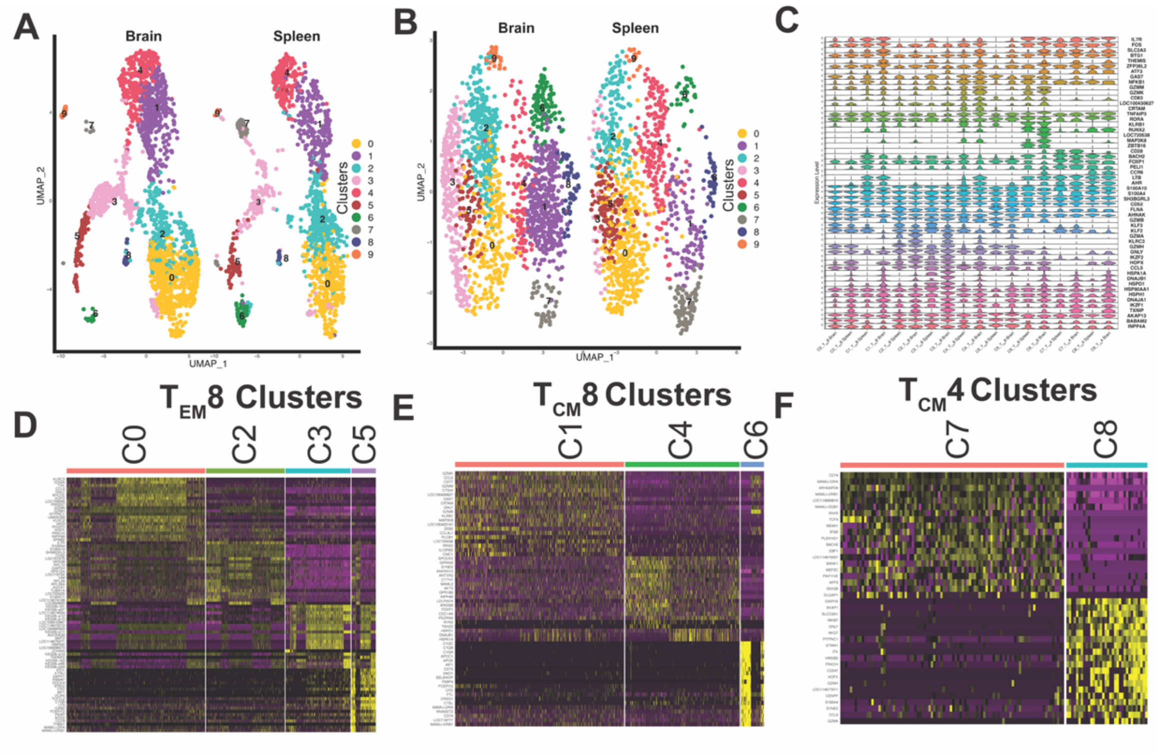

Figure S3. Single-cell transcriptomic analyses of CD45+ immune cells reveal presence of core T cell molecular programs in brain, related to Figure 1. (**A**) UMAP of scRNA seq transcriptome profiles color coded according to assigned cell types by tissue- brain (2137 cells) and spleen (1569 cells). (**B**) UMAP of T cell subclusters color coded according to assigned cell types by tissue - brain (1619 cells) and spleen (1159 cells). (**C**) Expression of top marker genes in each T cell cluster. (**D-E**) heat maps of gene expression within clusters demonstrate differential expression across clusters.

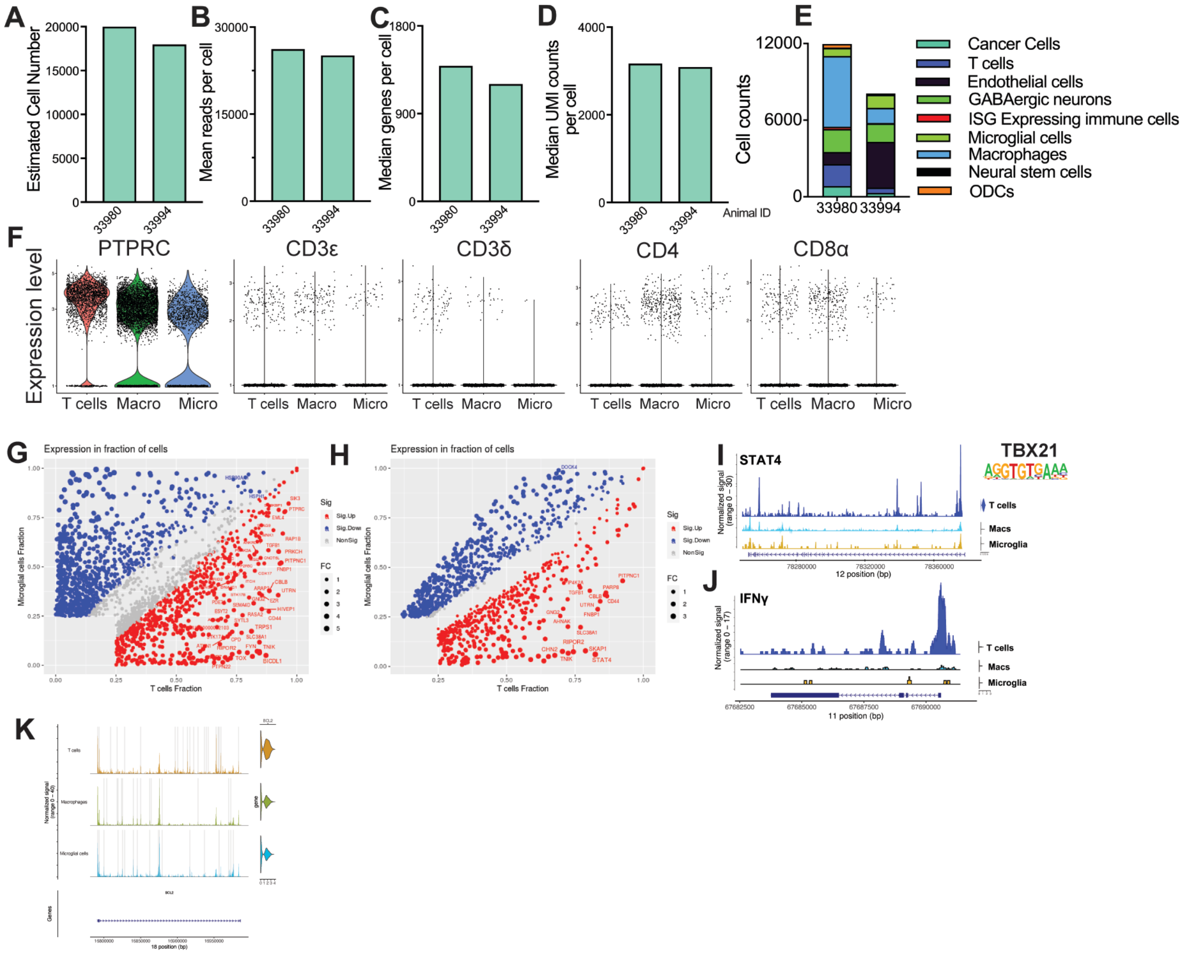

Figure S4. Single cell regulatory landscape of T cells and brain resident microglia, related to Figure 2. (**A**) lists cell numbers sequenced for each sample. (**B-D**) Mean, median reads per cell and UMI counts per cell. (**E**) Cell counts for each cluster shown per sample. (**F**) Expression profile of PTPRC shown in UMAPs and violin plots. Scatter plots show proportion and intensity of gene expression comparing (**G**) Microglia to T cells. (**H**) Macrophage to T cells. (**I-K**) Genomic regions showing snATAC-seq tracks of chromatin accessibility of STAT4, IFNγ, and BCL-2

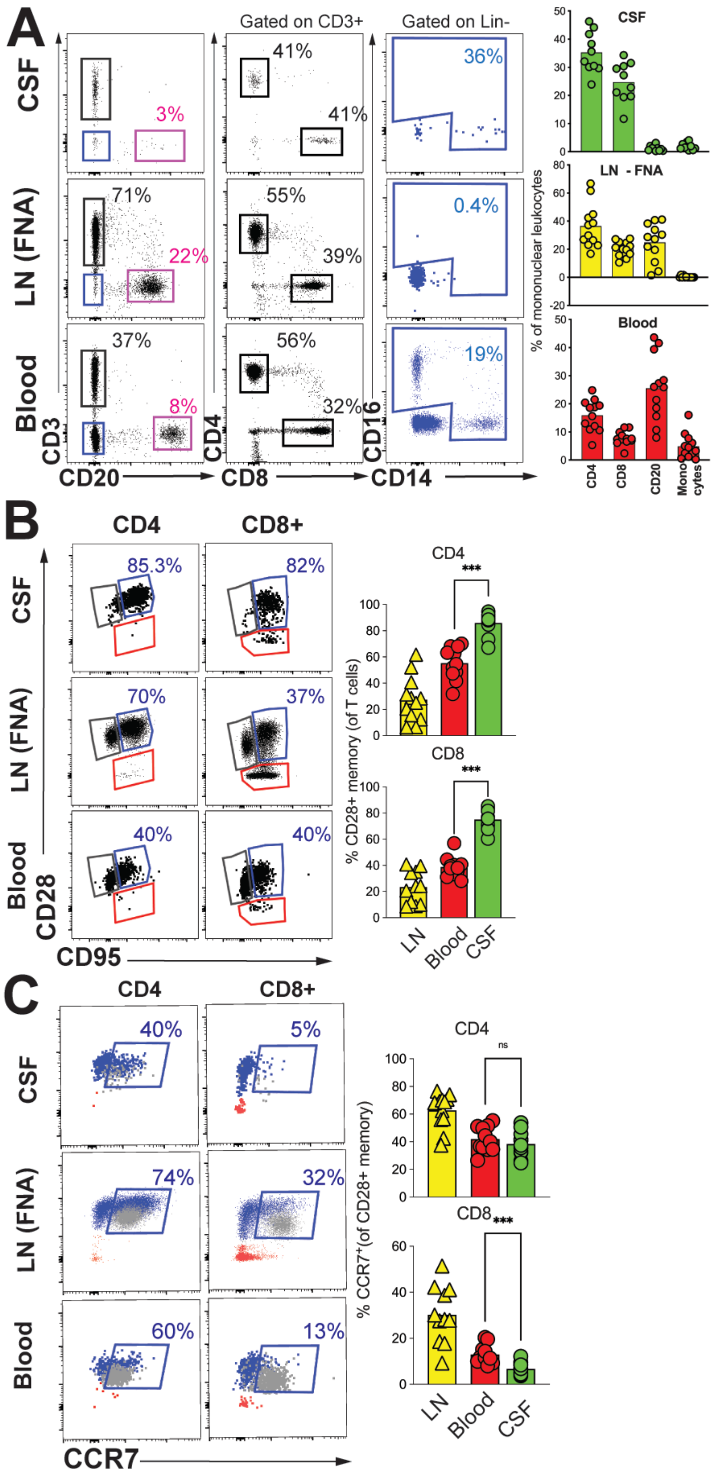

Figure S5. CCR7⁺ CD4 T cells in the CNS share phenotypic features with T_{CM} in blood and lymph nodes, related to Figure 3. (A) Representative flow plots identifying CD3 T cells, CD20 B cells, CD4 T cells, CD8 T cells and Monocytes (Left) and their frequencies (Right) in the CSF,

Lymph node Fine Needle Aspirate (LN FNA), and Blood of healthy rhesus macaques. CSF: n=10; LN-FNA: n =12; Blood: n=12 **(B)** Representative flow plots identifying expression of CD28 and CD95 on CD4 and CD8 T cells (Left) and their frequencies (Right) from the CSF, LN (FNA), and Blood. **(C)** Representative flow plots identifying CCR7 expression on CD28+ CD4 and CD28+ CD8 T cells (Left) and their frequencies in the CSF, Lymph node Fine Needle Aspirate (LN FNA), and Blood.

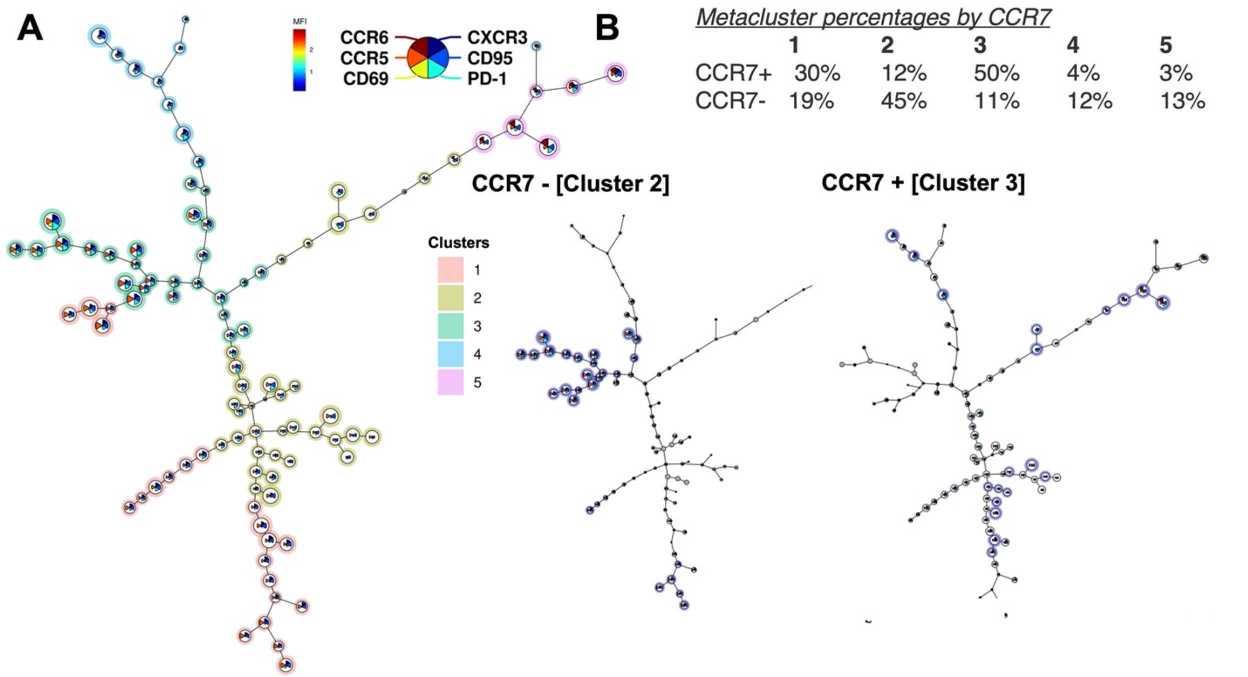

Figure S6. CCR7⁺ CD4 T cells in the CNS share phenotypic features with T_{CM} in blood and lymph nodes, related to Figure 3. (A) Minimal spanning tree constructed using the FlowSOM (self-organizing map) tool to map phenotypic relationships between live CD4 T cell isolated from healthy rhesus CSF. Star chart wedges denote expression of each indicated marker in cluster. Nodes indicate cellular cluster which are grouped into 5 larger Metaclusters indicated by color (metacluster 1: pink; metacluster 2; yellow; metacluster 3: green; metacluster 4: blue; metacluster 5: purple) which share similar phenotypes. Connections indicate phenotypically related cellular clusters. Nodes are represented as pie charts representing relative expression of chemokine and

activation markers (CCR6, CCR5, CD69, CXCR3, CD95, PD-1) within a given cluster. **(B)** Minimal spanning trees highlighting in blue, clusters that contain CCR7- (top) and CCR7+ (bottom) CD4 T cell populations.

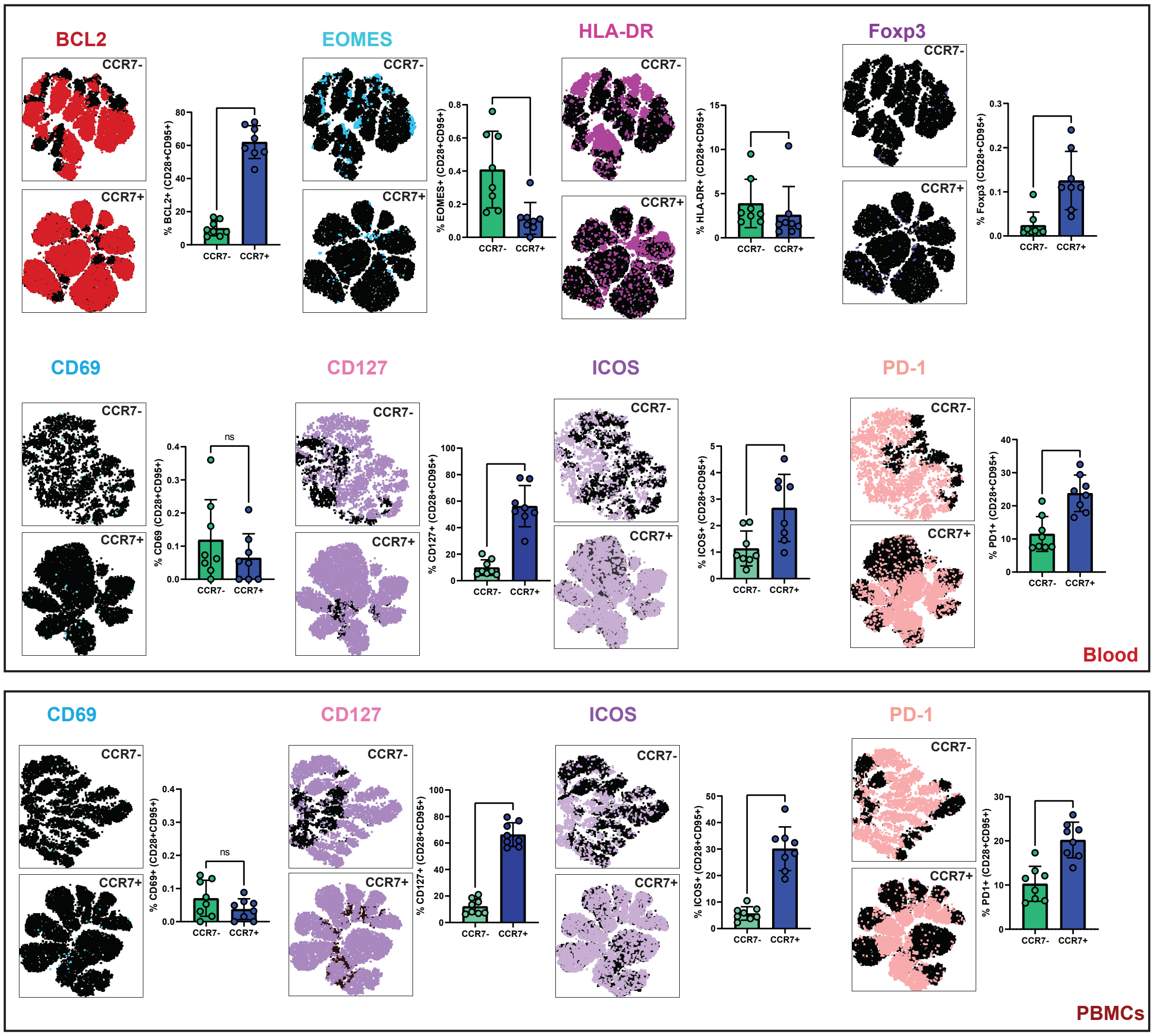

Figure S7. CCR7^{+} CD4 T cells in the CNS share phenotypic features with T_{CM} in blood and lymph nodes, related to Figure 3. Representative tSNE plot illustrating CD69, BCL2, CD69 (column 1), CCR5, EOMES, CD127 (Column 2), HLA-DR, ICOS (Column 3) , and CXCR3, Foxp3,

PD-1 (Column 4) expression on CD4+CD28+CD95+ CCR7-/+ T cells in the CSF, Blood and PBMCs; frequencies for each population are expressed to the right of the tSNE plots.

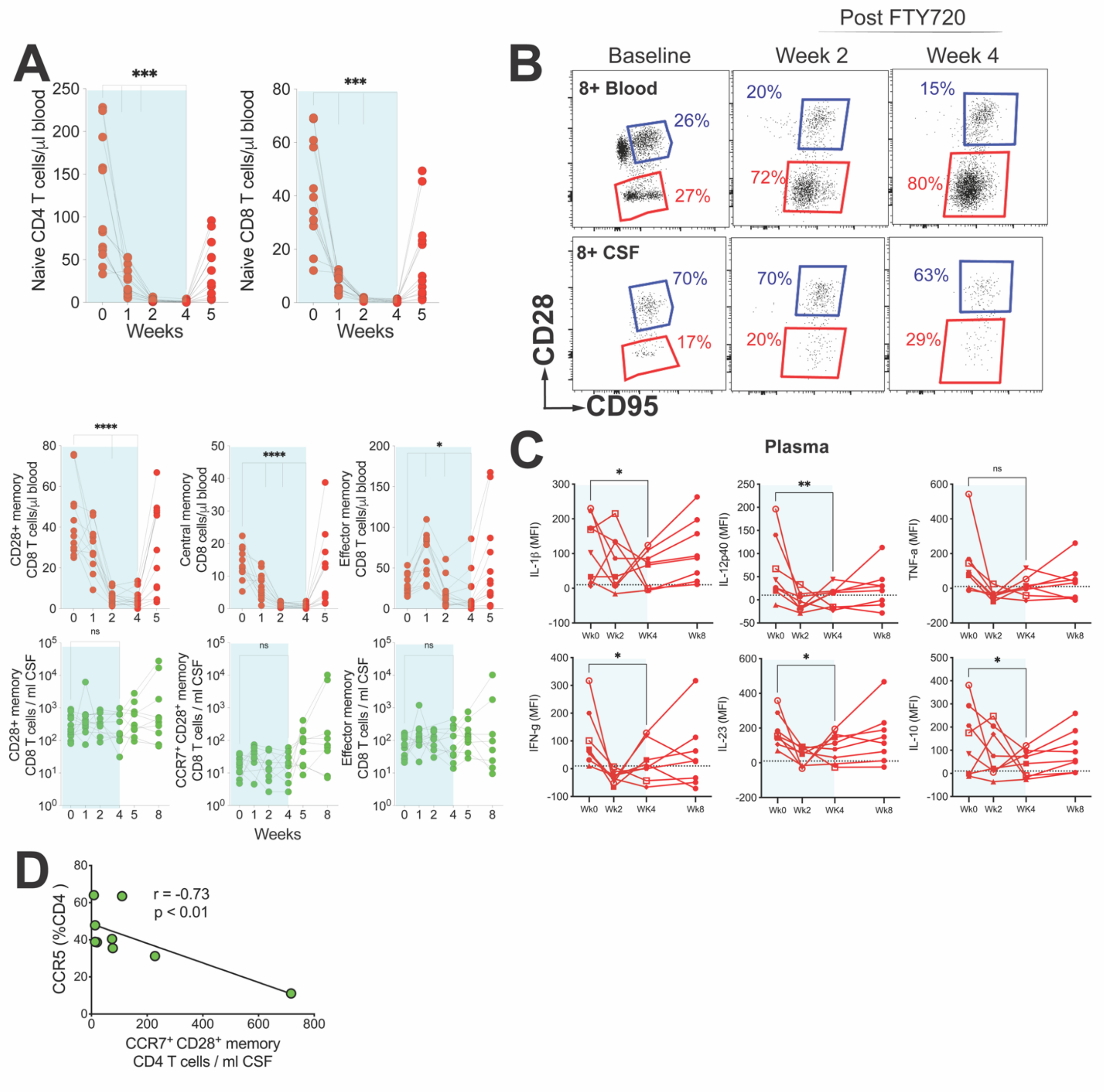

Figure S8. FTY720 mediated sequestration of CD4 T_{CM} in lymphoid tissues decreases CCR7⁺ CD4 T cell frequencies in CSF, related to Figure 4.(**A**) Frequencies of Naive CD4 T cells and Naive CD8 T cells in the blood over the course of the study (**B**) Representative longitudinal flow plots indicating CD28 and CD95 expression on CD4 T cells from the blood (top row) or the CSF (bottom row) (Left); Frequencies of CD28+ memory CD8 T cells, Central Memory

CD8 T cells, CCR7+CD28+ memory CD8 T cells, and Effector CD8 T cells in the blood and CSF over the course of the study. (**C**) Plasma levels of analytes over the course of FTY720 treatment. (**D**) Inverse correlation between CCR5+ CD4 T cell frequencies and absolute counts of CCR7+ CD4 T cells.

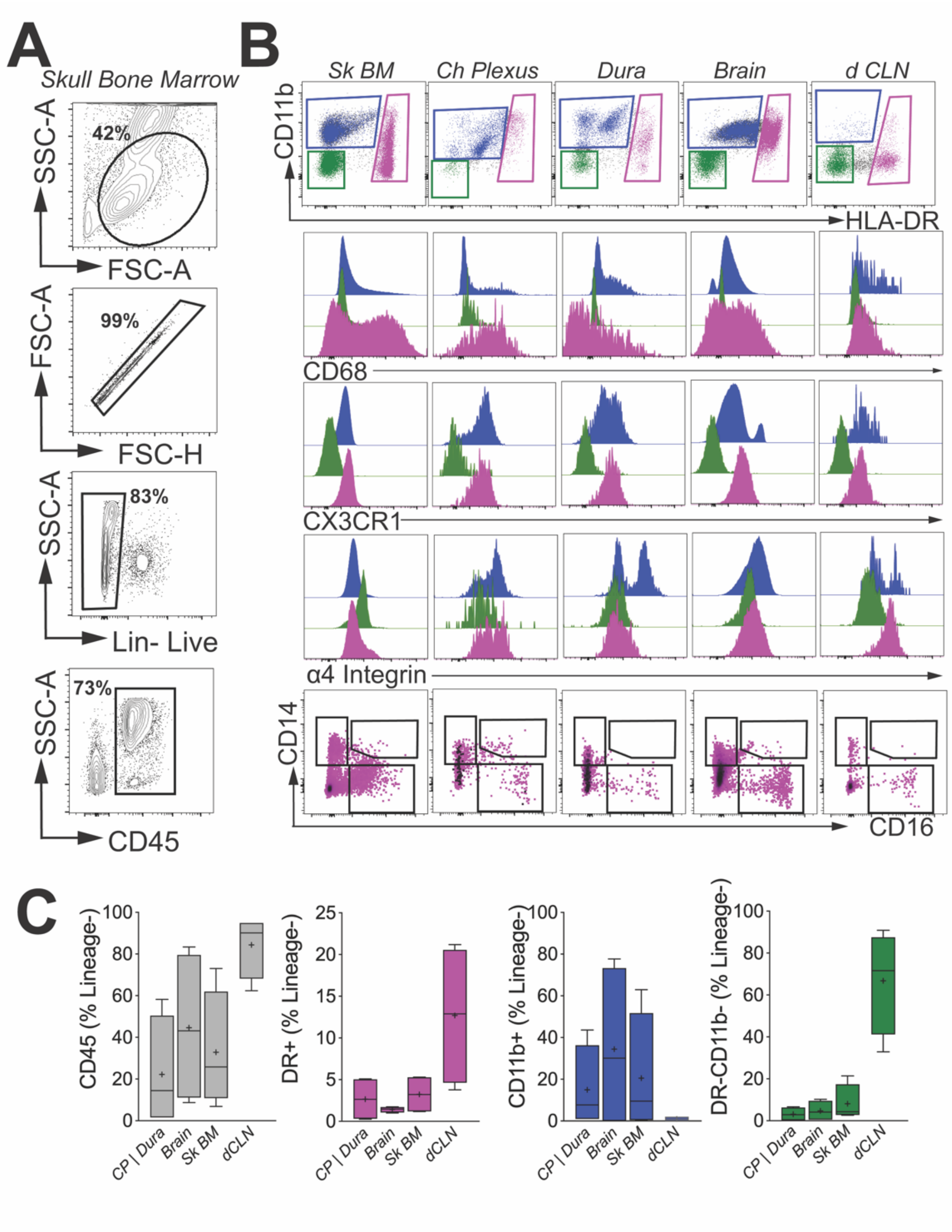

Figure S9. CCR7+ CD4 T cells in CNS exhibit functional T_{CM} features and reside within skull BM, related to Figure 5. Frequencies of Lineage (CD20/CD3/Dead) negative CD45+ cells (gray), MHC-II+ (DR) (pink), CD11b+ (blue), and DR-CD11b- cells within the choroid plexus (CP)/dura mater (Dura), brain, skull BM (Sk BM), and draining cervical lymph node (dCLN). Box and whisker plots indicate medians with quartiles.

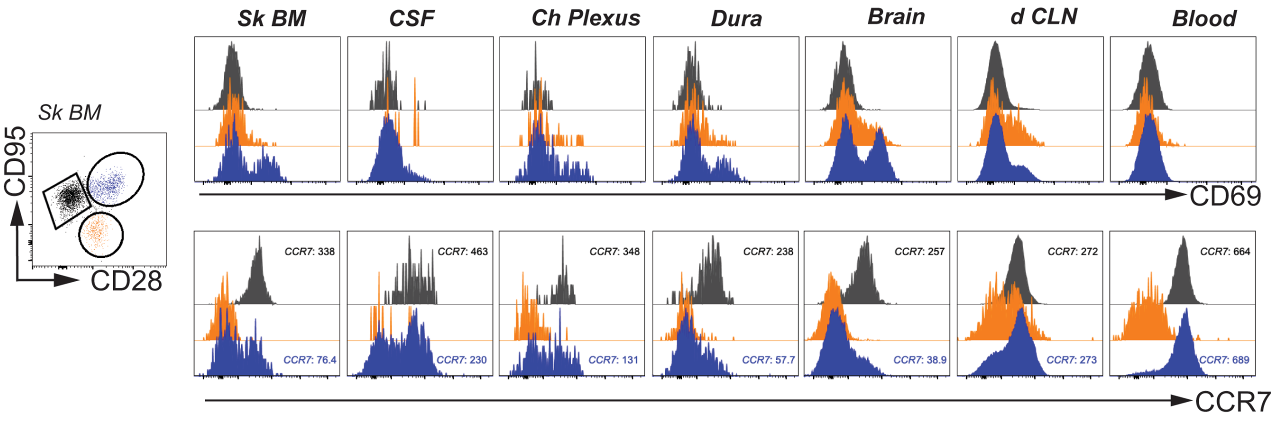

Figure S10. CD28+ CD4 T cells exhibit heterogeneity in the expression of CCR7 and CD69 in T_{CM} cells across the tissues, related to Figure 5. Representative flow plot of CD4+; CD95+CD28- (black), CD95+CD28+ (blue), and CD95-CD28+ (orange) cells from the skull BM; expression of CD69 and CCR7 shown by histogram for all 3 CD4 T cell subsets in the Skull BM, CSF, Choroid Plexus, Dura, Brain, Deep cervical lymph node, and blood.

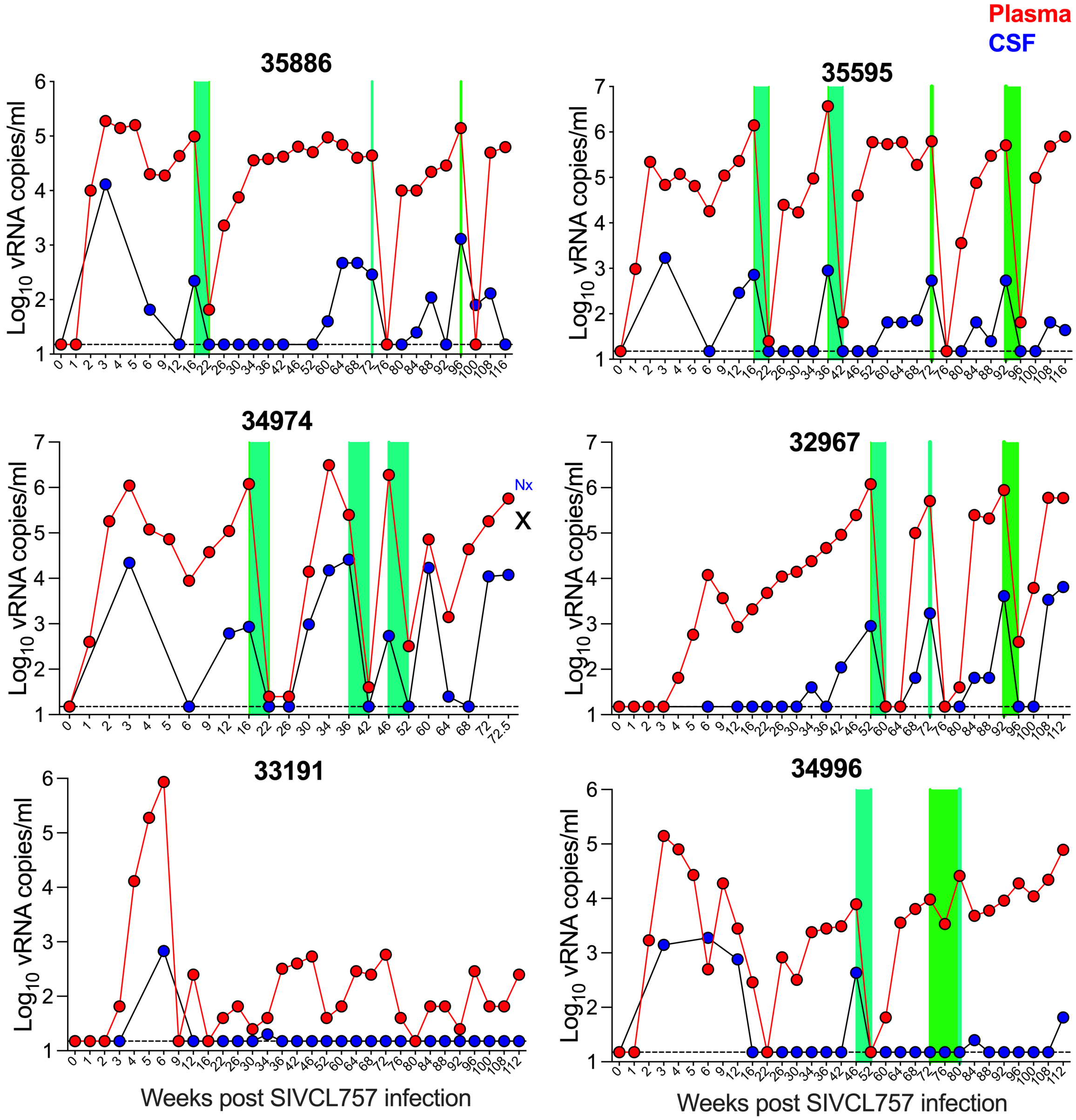

Figure S11. Longitudinal Plasma and CSF viral loads, related to Figure 6. Kinetics of vRNA in Plasma (red line) and CSF (blue) following SIVCL757 infection. Green bars indicate timing of antiretroviral therapy.

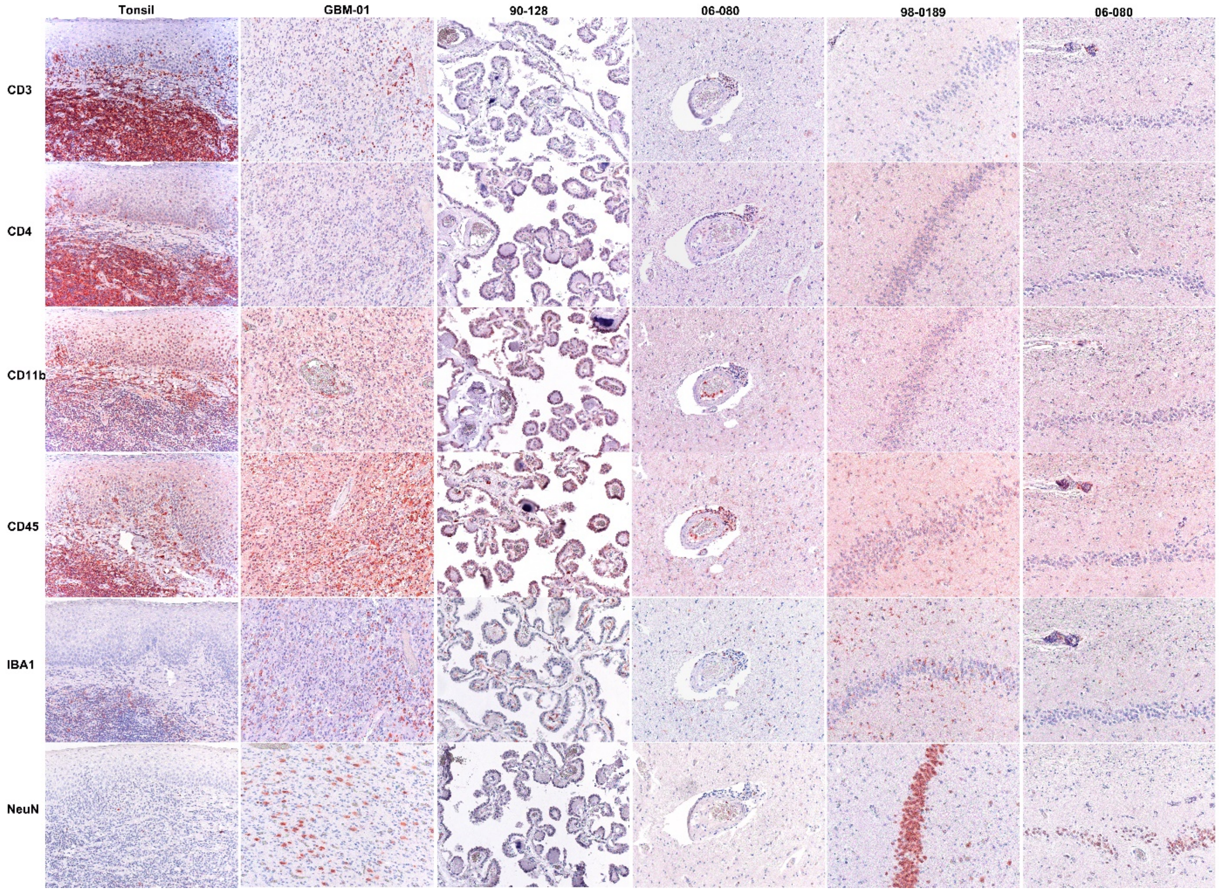

Figure S12. IHC reveals presence of T cells within the human brain, related to Figure 7.

T lymphocytes are localized to blood vessel in the non-demented human brain. H&E with immunohistochemical staining of lymphocyte specific markers (CD3, CD4), myeloid/leukocyte specific markers (CD11b, CD45, IBA1) and neuron-specific markers (NeuN) from paraffin embedded human tonsil and brain tissues derived from either a patient with glioblastoma (Sample ID: GBM-01) or non-demented patients (Sample IDs: 90-128, 06-080, 98-0189, 06-080). Tissue/patient samples are organized by columns and immunohistochemical markers by row.

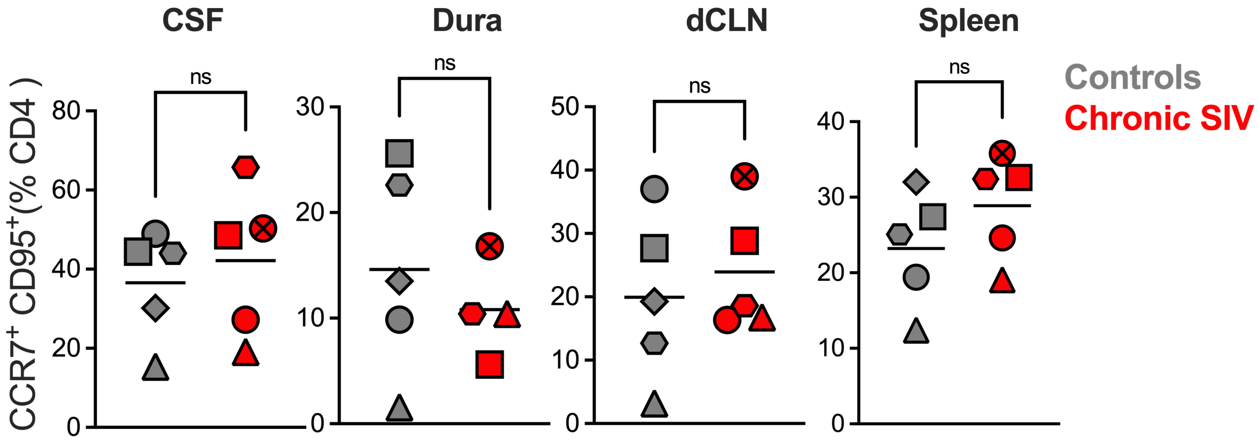

Figure S13. Preferential depletion of parenchymal CCR7+ CD4 T cells in chronic SIV infection, related to Figure 8. CCR7+ memory T cells in control (grey) and Chronic SIV infected (red) in the CSF, Dura, DCLN, and Spleen (left to right).
